# Exploring synthetic controls in rare diseases with a proof of concept in spinal cord injury

**DOI:** 10.1186/s12916-025-04405-3

**Published:** 2025-10-24

**Authors:** Louis P. Lukas, Samuel Håkansson, Miklovana Tuci, Abel Torres-Espín, Rüdiger Rupp, Olga Taran, Norbert Weidner, Fred Geisler, Martin Schubert, Frank Röhrich, Yorck B. Kalke, Rainer Abel, Doris Maier, Harvinder S. Chhabra, Thomas Liebscher, EMSCI study group, John L. K. Kramer, Marc Bolliger, Armin Curt, Catherine R. Jutzeler, Sarah C. Brüningk

**Affiliations:** 1 Department of Health Sciences and Technology (D-HEST), ETH Zurich, Zurich, Switzerland; 2 SIB Swiss Institute of Bioinformatics, Lausanne, Switzerland; 3 Spinal Cord Injury Center, University Hospital Balgrist, University of Zurich, Zurich, Switzerland; 4 School of Public Health Sciences, University of Waterloo, Waterloo, Canada; 5 Department of Neurological Surgery, University of California San Francisco, San Francisco, USA; 6 Department of Physical Therapy, University of Alberta, Alberta, Canada; 7 Faculty for Medicine, Heidelberg University, Heidelberg, Germany; 8 Spinal Cord Injury Center, Heidelberg University Hospital, Heidelberg, Germany; 9 University of Saskatchewan, Saskatoon, Canada; 10 Spinal Cord Injury Center, BG Klinikum Bergmannstrost Halle, Halle, Germany; 11 Spinal Cord Injury Center Orthopaedic Department, Ulm University, Ulm, Germany; 12 Spinal Cord Injury Center, Bayreuth, Germany; 13 Spinal Cord Injury Center, Trauma Center Murnau, Murnau, Germany; 14 Department of Spine and Rehabilitation, Sri Balaji Action Medical Institute, New Delhi, India; 15 Treatment Centre for Spinal Cord Injuries, Trauma Hospital Berlin, Berlin, Germany; 16 International Collaboration On Repair Discoveries (ICORD), University of British Columbia, Vancouver, Canada; 17 Department of Anesthesiology, Pharmacology, and Therapeutics, Faculty of Medicine, University of British Columbia, Vancouver, Canada; 18 Hugill Centre for Anesthesia, University of British Columbia, Vancouver, Canada; 19 Department of Radiation Oncology, Inselspital, Bern University Hospital and University of Bern, Bern, Switzerland

**Keywords:** Machine learning, Recovery prediction, Clinical trial design

## Abstract

**Background:**

Successfully completing clinical trials for rare and heterogeneous disorders, like spinal cord injuries (SCI), remains challenging, thereby reducing the ability to test and translate promising preclinical findings. We propose synthetic controls, derived from data-driven predictions of recovery in patients undergoing standard treatments, to mitigate these challenges, in particular related to patient recruitment.

**Methods:**

Based on data from the European Multicenter Study about Spinal Cord Injury (EMSCI) and the Sygen trial, we construct synthetic controls from personalized predictions of neurological recovery of sequences of segmental motor scores. A total of six architectures (linear, tree, and deep learning models) are compared. We demonstrate the applicability of synthetic controls through a simulation framework modeling the randomization process in a clinical trial and a case study that re-evaluates the recently completed Nogo Inhibition in SCI (NISCI) trial as a single-arm trial post hoc.

**Results:**

The primary dataset included 4196 patients from EMSCI and 587 patients from the Sygen trial for external validation. We identified a convolutional neural network as the best-performing architecture to predict segmental motor score sequences, achieving a median root mean squared error below the neurological level of injury of 0.55. Our trial simulations demonstrate that synthetic controls are a viable alternative to randomization, as the proposed solution reduces intercohort heterogeneity and leads to no significant differences with randomized controls in our case study reassessing a clinical trial.

**Conclusions:**

We provide a comprehensive benchmark of data-driven prediction architectures for neurological recovery after SCI. Apart from offering individual patients a specific recovery prediction, these models constitute the basis for synthetic controls. Using real-world data from a completed trial in SCI, we show that synthetic controls could mitigate the challenges of small cohorts and patient recruitment in rare disorders, offering the opportunity to maximize the number of patients receiving an investigative treatment.

**GitHub repository:**

https://gitlab.ethz.ch/BMDSlab/publications/sci/sci-in-silico-trials.

**Supplementary Information:**

The online version contains supplementary material available at 10.1186/s12916-025-04405-3.

## Background

Acute traumatic spinal cord injury (SCI) causes severe motor, sensory, and autonomic impairments through direct mechanical trauma and subsequent secondary pathophysiological processes [1]. Currently, no effective interventions, pharmacological or otherwise, are approved for restoring the damaged cord. The resulting lifelong deficits associated with SCI greatly impact the quality of life for affected patients, their caregivers, and society [2, 3]. With fewer than 250 cases registered per million inhabitants per year [2], SCI is considered a rare disorder and is hallmarked by extensive heterogeneity in its clinical presentation and recovery. Conducting randomized clinical trials (RCTs) to investigate novel treatments for rare diseases, including SCI [4], is highly challenging. Several regulatory-led initiatives support the development of innovative trial designs to address these challenges [5].

For instance, historical controls, that is, patient data from previous trials and existing databases, have recently been used in the Nogo Inhibition in SCI (NISCI) trial (ClinicalTrials.gov #NCT03935321) [6]. Synthetic controls further advance this concept. Here we define synthetic controls as predictions made using statistical [7] or data-driven models [8], trained on data external to a trial [9], which provide a counterfactual for the individual patient in the absence of treatment. Machine learning (ML) models, especially those representing universal function approximators, are flexible to draw relationships between features and output variables in the absence of a mechanistic understanding of this relationship [10]. Given the large variety of implementations, a comparison of potential models for deriving synthetic controls is key. A recent review highlighted that the use of ML models in SCI leans towards simplistic architectures, such as linear or tree-based models [11]. While these approaches are interpretable, they overlook dependencies specific to SCI data. For example, the sequential nature of segmental motor and sensory scores is often ignored and not accounted for, as statistically independent features are assumed in most models used in SCI [11]. For synthetic controls to be applicable, all information relevant to calculate the trial’s specific evaluation endpoint has to be predicted. In SCI, aggregate neurological recovery endpoints derived from segmental motor scores are particularly popular [12], implying that a model for synthetic controls should provide full segmental motor score information. Importantly, recent work has highlighted the relevance of predicting segmental motor recovery for an improved understanding of recovery [13] and the feasibility of deriving such predictions at the level of individual patients [14]. Such an approach differs from methods currently used, such as URP-CTREE [15], which provide outcomes using the average for groups instead of separate predictions for each individual.

Both historic and synthetic controls aim to maximize the number of individuals receiving investigative treatments through single-arm trials. The European Medicines Agency (EMA) recently recognized the potential of single-arm trials for rare conditions, acknowledging their suitability as a tool to provide evidence and support market approval for new treatments [16]. To conduct a single-arm trial, the EMA stresses the critical role of external information as either general knowledge about the natural course of the disease or clinical data derived from external sources (e.g., patient registries, observational studies). If a synthetic control is used, the description of the controls should be precise and made a priori. Further, it is essential to demonstrate that synthetic controls provide an unbiased and precise estimation of a treatment effect.

This study investigated the hypothesis that synthetic controls, generated by data-driven recovery predictions of segmental motor scores, are a viable control option to evaluate treatment efficacy in single-arm clinical trials in SCI. We benchmarked models of increasing complexity for generating synthetic controls and explored which features models rely on to drive the recovery prediction of a specific myotome by interpretability analysis. We compared synthetic controls to randomized controls using a simulation framework to quantify the impact of these methods for different cohort compositions. Finally, we illustrate a potential application through a case study using data from the recently completed NISCI trial.

## Methods

### Patient cohorts

This analysis uses data from the European Multicenter Study about Spinal Cord Injury (EMSCI, ClinicalTrials.gov #NCT01571531) collected between 2002 and 2023 in accordance with the Declaration of Helsinki across 33 centers. The review boards of all contributing centers approved the data collection. EMSCI patients undergo an International Standards for Neurological Classification of SCI (ISNCSCI) assessment [17, 18] at multiple time points throughout their recovery trajectory. Data of patients were included if at least one ISNCSCI assessment was available within 98 days after injury (DAI) in addition to an assessment at least 150 DAI. Patients with more than one assessment in these time windows were included as multiple instances for model training. Patients were further required to have information on injury severity classified by the American Spinal Injury Association (ASIA) Impairment Scale (AIS) and the neurological level of injury (NLI). We excluded patients with clear signs of deterioration (any motor score deterioration by two or more points between early and late assessments) because patients with traumatic SCI typically show an improvement or no change in motor and/or sensory scores over time. A deterioration of sensorimotor function would be related to the incidence of complications, which negatively impact this process. As EMSCI, the primary source of data for this study, does not record the time or kind of events which could result in deviations from such a severity-dependent typical recovery trajectory, it is not possible to model their effect on recovery, and cases presenting such non-explainable deterioration were excluded. Further, individuals with an initial NLI at or below S1 or an initial injury severity grade of AIS E were excluded. We used data obtained from the Sygen trial conducted between 1992 and 1998 in the United States for external validation in our benchmark [19]. Our case study (Sections 2.6 and 3.5) is based on the European NISCI trial completed in 2023 [6]. Additional details on all datasets can be found in Additional File 1: Section 1.1.

### Input data

The ISNCSCI assessment provides the best balance between data availability and the detailed information necessary to justify its application for synthetic control generation. Full ISNCSCI assessments comprising segmental motor scores (MS), light touch scores (LTS), and pinprick scores (PPS), AIS grade (categorical: A-D), voluntary anal contraction (VAC; absent or present), deep anal pressure (DAP; absent or present), and NLI were included. We included patient age at the time of injury (years), sex, and the time of early and late assessments (DAI) as covariates of interest. For additional information on the ISNCSCI assessment and handling of missing values, refer to Additional File 1: Section 1.2.

### Outcome variable

Following popular SCI clinical trial endpoints [20], our analysis focuses on motor recovery. Specifically, we predict all segmental motor scores from the ISNCSCI examination, a nuanced perspective on recovery compared to aggregate metrics such as upper extremity motor score (UEMS) or lower extremity motor score (LEMS) [21].

### Recovery prediction model benchmark

**Fig. 1:**
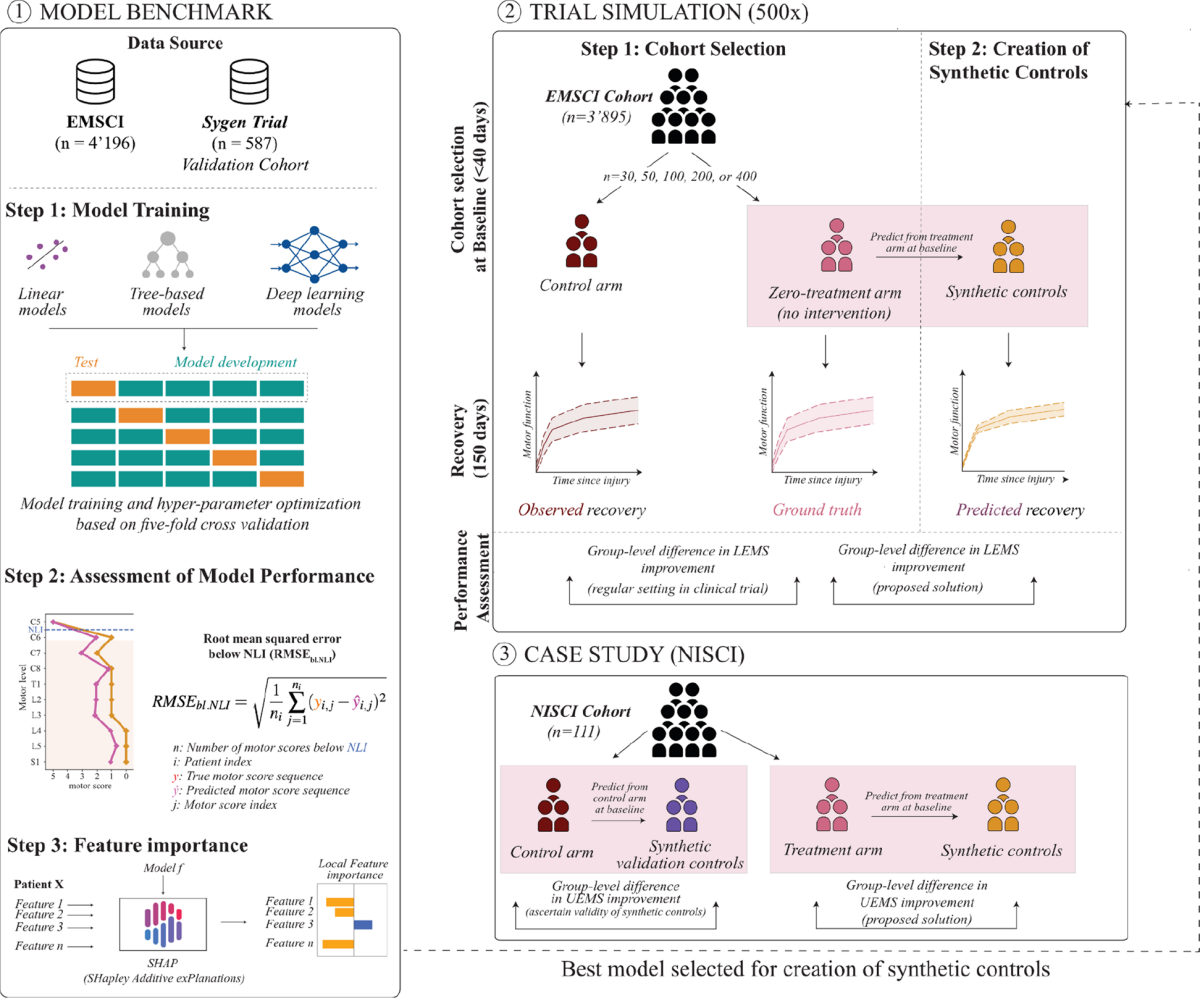
Overview of the study design. **1** Model benchmark. The EMSCI dataset is used for model training and testing based on ISNCSCI assessments (acute and recovery phase). Models predict segmental motor scores at recovery. Sygen data was used for external validation. Different prediction models are trained using fivefold cross-validation and evaluated using the RMSE_bl.NLI_. Variable importance is analyzed. **2** Trial simulations with synthetic controls. The best-performing model is used to create synthetic controls, i.e., a prediction of patient recovery in the absence of treatment. We randomize patients into two groups, *control* (dark red) and *zero-treatment* (pink). We derive two outcomes for the “*zero-treatment*” group: observed recovery which reflects natural recovery, and predicted recovery corresponding to the proposed *synthetic control* mechanism (yellow)*.* We compare the *zero-treatment* group recovery to the two types of controls based on LEMS improvement differences. This process is repeated 500 times. **3** NISCI case study. We test the applicability of the synthetic controls using data from the recently completed NISCI trial and compare recovery derived from synthetic controls in both control and treatment arms with observed recovery in the control arm

Six recovery prediction models were implemented (Fig. 1, panel 1). We tested regularized linear regression, random forest, and extreme gradient-boosted trees, given their popularity in SCI research [11] and ease of implementation [22]. We further implement three types of deep learning approaches targeting sequence information: (i) convolutional neural networks (CNN), which learn sequential dependencies through filtering operations; (ii) sequence-to-sequence transformers, which convert an acute injury-phase sequence of ISNCSCI scores to a recovery phase sequence; (iii) graph neural networks (GNN), which allow us to explicitly specify relations inherent to a sequence (Additional File 1: Fig. S1). To quantify the contribution of individual features to the prediction outcome, we calculate feature importance rankings using SHapley Additive exPlanations (SHAP) [23], which perturbs the input data and inspects whether the output of the model changes to estimate feature importance. Detailed information on model implementations, hyperparameters, and model interpretation is provided in Additional File 1: Sections 1.3.1 to 1.3.4 [14, 24–31] and Additional File 1: Tables S1 and S2.

#### Performance evaluation

Prediction performance is quantified by the root mean squared error (RMSE) between true and predicted segmental MSs below the initial NLI (RMSE_bl.NLI_). RMSE measures the average difference between values predicted by a model and the actual values. RMSE below the initial NLI allows for comparison across patients independent of their NLI. Results are reported for held-out data (test sets for EMSCI in a cross-validation setting, Sygen as external) with uncertainty quantified based on perturbations of the input from estimates of the ISNCSCI assessment uncertainties. Detailed performance evaluation is reported in Additional File 1: Section 1.3.5.

### Trial simulation framework

To compare synthetic and randomized controls, we simulate both scenarios in the absence of an investigative treatment (Fig. 1, panel 2). Initially, we quantify the potential differences in natural recovery between two groups occurring only due to the stochastic effects of the patient randomization process and the variability of recovery following SCI. To this end, we establish a control group by randomly drawing individuals with replacement from the EMSCI data. For comparison, a “*zero-treatment*” group is established by identifying individuals in EMSCI who are as similar as possible to the individuals in the control group. “*Zero-treatment*” reflects the fact that individuals in this group would receive an investigative treatment in an RCT, but here only represent natural recovery due to EMSCI’s observational nature. Hence, an average treatment effect of zero is expected in comparison with the control group. This setup represents an ideal outcome of the randomization process, resulting in similar distributions of covariates and neurological assessment results at baseline. Details on the steps of the simulation process are provided below.

*Cohort randomization*: For a specified number of patients, inclusion criteria were the time of assessment (acute and recovery phase), injury severity distribution (AIS grades), and extent of injury (para- and tetraplegia). To ensure comparable group-level statistics, individuals in the “*zero-treatment* group” are matched with respect to age (± 5 years), sex, NLI (± 2 segments), and AIS grade to the *control group*.

*Outcome simulator*: We analyzed (i) randomized controls (*observed* recovery of the *control group*), and (ii) synthetic controls (*predicted* recovery given the baseline characteristics of the patients in the “*zero-treatment*” group).

*Evaluation endpoint*: We scored group-level differences between the “*zero-treatment*” group and either the *randomized* or *synthetic control* group on the mean LEMS improvement (ΔLEMS_impr._) computed from baseline to follow-up, which reflects neurological recovery.

We complete simulations on a subset of the EMSCI cohort (see *Patient cohorts*) comprising patients with an initial assessment no later than 40 DAI. Each patient is considered with their earliest (baseline) and latest (recovery) ISNCSCI assessments. We repeated these simulations 500 times to account for the stochastic nature of the randomization and matching processes, and visualized the distributions of ΔLEMS_impr._ as boxplots. Group-level differences should be close to zero (ΔLEMS_impr._ ≈ 0) as no treatment is provided, and the effect size equals zero accordingly.

### Case study: NISCI trial

We illustrate a single-arm trial protocol enabled by synthetic controls on the NISCI trial. This trial, completed in 2023, aimed to determine the effectiveness of Nogo-Inhibition after cervical SCI [6]. We quantify the potential benefits and limitations of moving towards a single-arm trial using synthetic controls by performing a comparison of group sizes required in a standard RCT and a single-arm trial with synthetic controls. For this purpose, we consider the outcome as defined for the NISCI trial, UEMS improvement between follow-up (168 DAI ± 7 DAI) and baseline (≤ 28 DAI), UEMS_impr_ in cervical injury patients with baseline UEMS of less than 29 points. Importantly, the use of synthetic controls implies a paired *t*-test to evaluate the outcome, while the use of two distinct groups requires a two-sample *t*-test. We assess the validity of the synthetic controls as counterfactuals by comparing the outcome of interest (UEMS_impr_) derived from synthetic and randomized controls. We subsequently compare the estimated group sizes required for a range of treatment effects in both scenarios. Further details of the retrospective analysis are provided in Additional File 1: Section 1.4.

## Results

### Patient cohort description

From 6607 patients in the EMSCI dataset, 4196 patients (12,796 instances) met our inclusion criteria for the ML benchmark and 3895 patients for the trial simulations (Additional File 1: Fig. S2). From Sygen, 587 patients (2718 instances) were included (Additional File 1: Fig. S3). Finally, from NISCI, 111 patients were included. Table 1 summarizes these four cohorts (see Additional File 1: Table S3 for details). In comparison to EMSCI and NISCI, Sygen patients were more severely injured (AIS A: 43.1%, 26.7% vs 64.2%), assessed earlier, and younger at injury (mean age (SD): 46.3 (18.3), 45 (17) vs 32.0 (13.2)).

**Table 1:** Characteristics of EMSCI, Sygen, and NISCI cohorts. Values reported at patient level and for earliest initial and outcome assessment; cohort analyzed for EMSCI includes imputed values (Additional File 1: Tables S3 to S6). EMSCI cohorts for the machine learning benchmark and trial simulations differ in the cut-off for the latest possible time allowed for the earliest assessment (benchmark: ≤ 98 DAI; trial simulations: ≤ 40 DAI) and follow-up time reported (benchmark: ≥ 150 DAI; trial simulations: last available follow-up). Distributions of age and initial injury severity were compared using a two-sided *t*-test and a chi-squared test respectively with a significance level 0.05. ^*^ and ^#^ indicate statistically significant differences between EMSCI and Sygen, and EMSCI and NISCI cohorts respectively

		**EMSCI**		**Sygen**	**NISCI**
		**Benchmark** (*n* = 4196)	**Trial simulations** (*n* = 3895)	**Benchmark** (*n* = 587)	**Benchmark** (*n* = 111)
**Age (years)**, mean (SD)		46 (18)^*^	46 (18)	32 (13)^*^	46 (17)
**Time to first assessment (days)**, mean (SD)		22 (20)	18 (12)	9 (15)	21 (5)
**Time to (first) follow-up (days)**, mean (SD)		216 (91)	301 (104)	209 (55)	190 (18)
**Sex (female)**, *n* (%)		928 (22.1)	859 (22.1)	122 (20.8)	16 (14.4)
**Initial VAC (present)**, *n* (%)		1280 (30.5)	1174 (30.1)	87 (14.8)	45 (40.5)
**Initial DAP (present)**, *n* (%)		2197 (52.4)	2048 (52.6)	202 (34.4)	77 (69.4%)
**Initial neurological level of injury**, *n* (%)					
Cervical		2256 (53.8)	2064 (53.0)	448 (76.3)	111 (100)
Thoracic		1563 (37.3)	1475 (37.9)	139 (23.7)	0
Lumbar		377 (9.0)	356 (9.1)	0 (0.0)	0
Sacral		-	-	-	-
**Initial Injury severity (AIS grade)**, *n* (%)					
AIS A		1807 (43.1)^*,#^	1675 (43.0)	377 (64.2)^*^	31 (27.9)^#^
AIS B		539 (12.5)^*,#^	501 (12.9)	64 (10.9)^*^	23 (20.7)^#^
AIS C		761 (18.1)^*,#^	721 (18.5)	118 (20.1)^*^	28 (25.2)^#^
AIS D		1089 (26.0)^*,#^	998 (25.6)	28 (4.8)^*^	29 (26.1)^#^

### Model benchmark

Table 2 summarizes the benchmark performance to identify the best model to provide synthetic controls. While all approaches yield relevant predictions (population median RMSE_bl.NLI_ < 1, Additional File 1: Table S7), the CNN consistently outperformed all other models (median RMSE_bl.NLI, CNN_ 0.55 on EMSCI, all times). Validation on the independent Sygen cohort confirms the ranking of the investigated models.

**Table 2:** Benchmark results as median RMSE_bl.NLI_ and (2.5%-ile, 97.5%-ile) on held-out data for EMSCI and Sygen (external validation). The best-performing approach (lowest RMSE_bl.NLI_, if tied on median lower value for 97.5%-ile better) is highlighted. Columns labeled *All times* include all instances. Columns labeled *4* ± *1 weeks* include instances with an initial assessment between 21 and 35 DAI

	**EMSCI**		**Sygen**	
	All times	4 ± 1 weeks	All times	4 ± 1 weeks
Linear regression	0.63 (0.00, 2.22)	0.64 (0.02, 2.15)	0.70 (0.00, 2.32)	0.68 (0.08, 2.15)
Random forest	0.61 (0.01, 2.17)	0.60 (0.01, 2.08)	0.64 (0.03, 2.37)	0.61 (0.03, 1.89)
XGBoost	0.61 (0.01, 2.12)	0.61 (0.01, 2.08)	0.70 (0.01, 2.33)	0.68 (0.02, 2.00)
CNN	**0.55** (0.00, 2.50)	**0.55** (0.00, 2.57)	**0.57** (0.00, 2.85)	**0.60** (0.00, 2.90)
Transformer	0.63 (0.00, 2.58)	0.63 (0.00, 2.57)	0.65 (0.00, 3.00)	0.71 (0.00, 3.27)
GNN	0.58 (0.00, 2.60)	0.60 (0.00, 2.47)	0.68 (0.00, 2.61)	0.63 (0.00, 2.57)

*Abbreviations*: *RMSE*_*bl.NLI*_ root mean squared error (RMSE) below neurological level of injury (bl.NLI), *CNN* convolutional neural network, *GNN* graph neural network

**Fig. 2:**
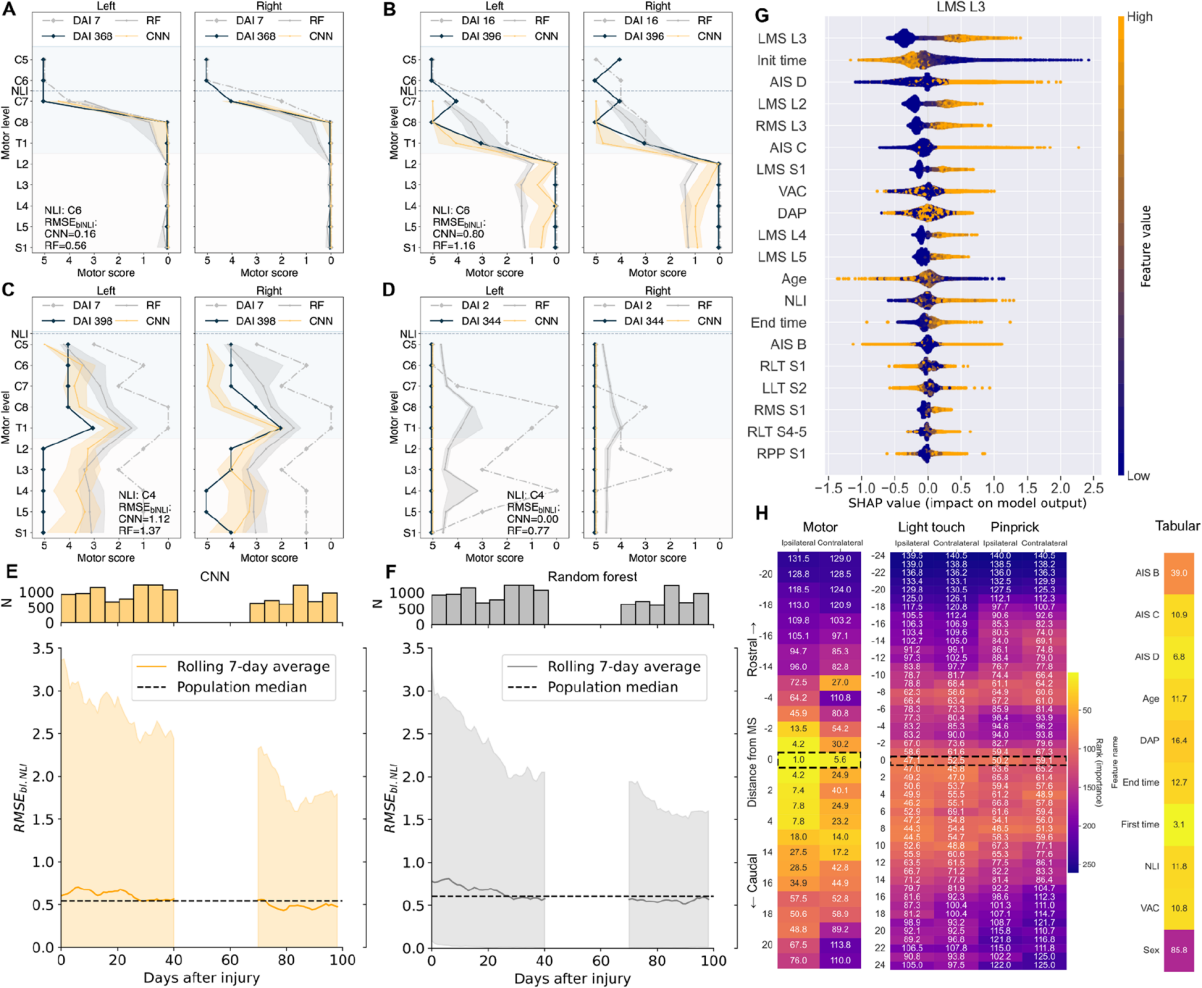
Model benchmark. **A**–**D** MS sequence predictions for representative patient examples of AIS grade A, B, C, and D achieving median RMSE_bl.NLI_ performance. The *y*-axis represents consecutive myotomes C5 to T1 (cervical spinal cord) and L2 to S1 (lumbar spinal cord), and the *x*-axis represents the motor scores. The plots show predicted (CNN in yellow, Random forest in gray), observed recovery (black), and initial (dashed gray) MS sequences. **E**, **F** RMSE_bl.NLI_ over time of initial assessment in DAI for CNN (**E**) and Random forest (**F**). The dashed line shows the 7-day rolling mean of the medians for each day and the shade is the 2.5th–97.5th percentile. **G** Feature importance example for CNN predictions of the left motor score L3. **H** Importance of the variables by proximity to the endpoint MS. Motor scores, light touch, and pinprick scores are colored by importance ranking and aggregated by distance from the endpoint MS. The dashed rectangles indicate the level of the endpoint MS

Figures 2A–D show representative patients with acute and recovery phase segmental motor scores together with predictions and prediction uncertainty for the best-performing CNN and a random forest for comparison. Importantly, we observed an improvement in prediction performance and a reduction in uncertainty stemming from input data as the assessment time moves further from the date of injury (Fig. 2E–F, Additional File 1: Fig. S4 and S5). Regarding patient subgroup performance (Additional File 1: Tables S8 and S9), we observe the best results in the most (AIS A) and least severely injured (AIS D) subgroups. Recovery from intermediate severity (AIS B, C) is more difficult to predict (median RMSE_bl.NLI_ AIS B 0.78–0.99, AIS C 1.16–1.38). Analyzing the mean of summed residuals below the NLI (Additional File 1: Tables S10 to S12) shows that all deep learning models have a median of 0, meaning no consistent over- or underestimation of motor scores, whereas linear and tree-based models consistently overestimate these.

### Which features associate with segmental motor recovery?

We evaluated the contribution of individual variables to the prediction of segmental motor recovery using SHAP values. A representative example of features contributing to the prediction of the left L3 motor score is shown in Fig. 2G: time of initial assessment and proximal motor scores are the most important. Figure 2H summarizes the contribution of all variables to the recovery of a myotome of interest (MS_endpoint_), represented as a relative distance of a contributing myotome and dermatome to MS_endpoint_. Overall, the importance of the MS variable increases with proximity to MS_endpoint_, with scores ipsilateral and caudal to MS_endpoint_ having a higher importance rank than those rostral. In addition to MS values, we identify initial assessment time, VAC, AIS grade, age, and NLI as important factors for predicting segmental motor recovery (Additional File 1: Fig. S6).

### Trial simulations of synthetic and randomized controls

**Fig. 3:**
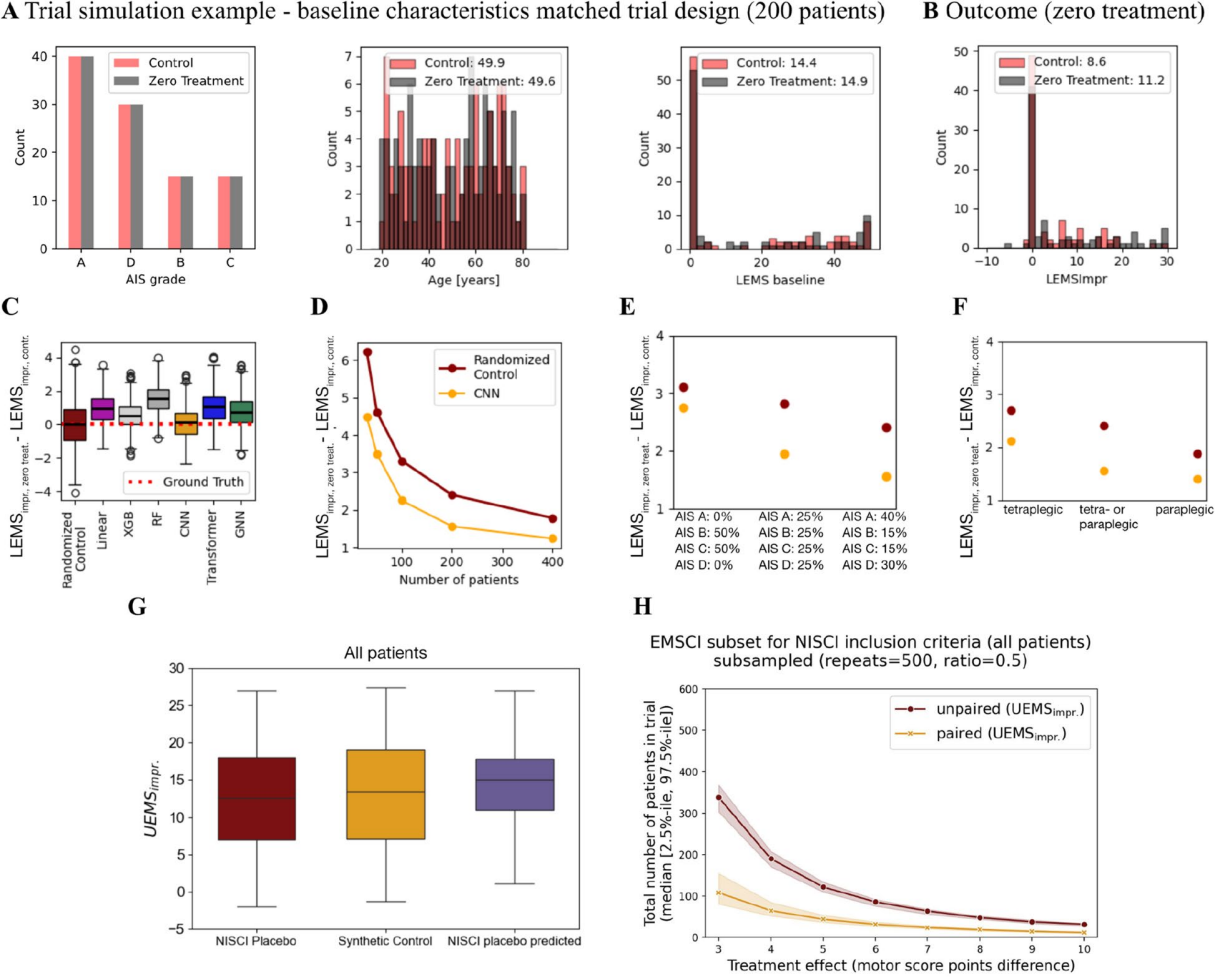
Trial simulations. **A** Baseline characteristics of a single simulated trial comprising 200 patients (paraplegia or tetraplegia) randomized into two groups. **B** Distribution of the patient-specific improvement in LEMS in the absence of any treatment yielding differences in the cohort means as indicated in the legend. **C** Corresponding overview of repeated trial simulations (500 trials) as indicated in **A**. The boxplot shows the group-level differences in mean LEMS_impr._ (∆LEMS_impr._) for randomized (leftmost, red box) and synthetic controls originating from different models shown in increasing order of computational complexity. **D**–**F** 95th percentile of the cohort LEMS improvement differences (P_95_(LEMS_impr, zero treat_ − LEMS_impr, contr._)) for trial simulations of increasing cohort size (**D**), varying AIS grade compositions (**E**), and varying plegia (**F**) for randomized and synthetic controls based on CNN predictions. All other trial simulation parameters correspond to the characteristics shown in **A**. **G** Outcome of interest (UEMS_impr._) in NISCI placebo and synthetic control groups illustrating the equivalence of the synthetic control to the placebo control on the primary outcome in the NISCI trial. **H** Repeated power calculations with relevant parameters on outcome (UEMS_impr._) computed from subset of EMSCI meeting NISCI inclusion criteria

We show a representative example of a single trial simulation for a clinically relevant cohort of 200 patients randomized into two groups matched by AIS grade distribution as observed in EMSCI, age, NLI, and sex. Figure 3A summarizes key characteristics of the groups at baseline. The corresponding recovery phase improvements in LEMS during standard treatment and rehabilitation show a mean 3.6-point difference at the group level (Fig. 3B) driven by the stochasticity of the patient selection. Figure 3C summarizes the results of 500 simulations using either randomized or synthetic controls generated by different models as the distributions of the group-level differences in LEMS improvement from baseline. Despite optimal cohort matching, randomized controls cover a range of possible trial outcomes, as indicated by the extent of the boxes and whiskers that reach far beyond the ground truth of the expected recovery, yielding no group-level differences. Synthetic control performance varies depending on the model used and may display systematic bias. A synthetic control based on the CNN was most centered around the ground truth expectation of zero (random control; median: − 0.05, 95th percentile 2.40, CNN; median: 0.16, 95th percentile 1.56) and narrower compared to randomized controls. Moreover, we show the superiority of synthetic controls also across different cohort sizes (Fig. 3D), AIS grade compositions (Fig. 3E), and patient characteristics (Fig. 3F). Here, the 95th percentile of the cohort LEMS improvement differences** (**P_95_(LEMS_impr, zero treat_ − LEMS_impr, contr._)) of the synthetic controls consistently falls below that of the RCT.

### Synthetic controls provide scope for alternative clinical trial design

Our retrospective analysis of the NISCI trial showed that a single-arm trial design, enabled by the use of synthetic controls, is feasible. We first validated the performance of the CNN predictions in a subset of EMSCI meeting NISCI inclusion criteria (Additional File 1: Table S13, Fig. S5), yielding a median RMSE_bl.NLI_ of 0.93 (0.14, 2.93).

When directly comparing synthetic and NISCI placebo controls (Fig. 3G), we do not observe significant differences in UEMS_impr_ (NISCI placebo (randomized control): 12.67 (1.00, 26.85); mean (2.5%-ile, 97.5%-ile); synthetic controls: 13.42 (0.13, 26.26); *p* = 0.610). Power calculations for the NISCI trial’s inclusion criteria showed that a single-arm trial could detect a treatment effect of five motor points based on 44 patients at a significance level of 0.05 and power of 0.8, while a trial using treatment and control groups would require a total of 122 patients (61 patients per group) (Fig. 3H). These findings are robust to variations in parameter estimates introduced by subsampling of the cohort from which these are derived (single-arm trial: 44 (37, 54), RCT: 122 (110, 134); median group size (2.5%-ile, 97.5%-ile)).

## Discussion

Clinical trials of rare disorders are inherently difficult. Traumatic SCI is a prime example, with low incidence and extensive heterogeneity with respect to lesion level, severity, and recovery. We highlight the resulting constraints of randomization in SCI trials. While these constraints could be addressed by increased statistical power of larger cohorts, it is important to point out that current clinical trials focus on small to intermediate cohort sizes, with 73% of trials reporting enrollment of 50 or fewer patients [32]. Increasing cohort sizes may not be feasible or would require extensive trial durations. Synthetic controls are a promising option in this setting [33], in particular for phase I and phase II trials, which commonly rely on smaller cohort sizes. However, an understanding of the impact of synthetic controls derived from prediction models was previously missing. Our study presents a comprehensive evaluation of several approaches for recovery prediction from neurological assessments in this context. Our study provides four key contributions: (i) We demonstrate the merit of incorporating the inherent segmental structure of the spinal cord in prediction models. (ii) Our variable importance and longitudinal analyses underscore the influence of predominantly caudal motor and sensory function on motor recovery. (iii) We offer a quantification of the minimum difference in outcome measure necessary for a clinical trial of a specific patient composition to demonstrate treatment efficacy, i.e., show a difference beyond what is expected by chance. (iv) We validate the performance of our synthetic controls compared to randomized controls using trial simulations and highlight the potential of applying synthetic controls through a case study based on a recently completed clinical trial in SCI.

Previous work has shown that the AIS grade and aggregated upper or lower extremity motor scores do not sufficiently represent motor function [34]. We take a step further and predict all segmental motor scores, which provide a more detailed representation of expected recovery and enable a better understanding of potential deviations from this expectation based on predictions of segmental scores. Additionally, the prediction of segmental motor scores enables researchers to derive a number of potential trial outcomes such as upper or lower extremity motor scores from a single model instead of requiring separate models for each of these outcomes. Our deep learning models, which account for the sequential dependence of the ISNCSCI exam scores, outperformed linear and tree-based models. External validation on the Sygen dataset confirmed the robustness of these models and the ranking of the best-performing models. As expected, performance varied between groups of patients stratified by injury severity: better results were observed at either end of the severity scale (AIS A, AIS D) compared to intermediate severities (AIS B, AIS C). Importantly, the aim of this study was to show the clinical value of predictions for AIS B/C despite their imperfection and to account for the inherent assessment uncertainties that are particularly large for these patients [35]. A strength of our study was the visualization of prediction uncertainty, which is crucial for increased insight and trust in the models during translation to clinical practice and decision-making [36].

Our feature importance analysis highlighted the impact of the timing of the initial assessment. Moreover, motor scores in proximity to the myotome of interest, particularly those caudal to it, were shown to outweigh those rostral, while motor scores generally precede sensory scores in importance. This observation aligns with previous findings [37–41]. The importance of the time of assessment is dictated by the sigmoidal recovery trajectory. Particularly in the early phase, a patient’s neurological status, as assessed through ISNCSCI, may improve notably, implying a need to account for recovery occurring until the time of the initial assessment. This also explains improved prediction performance for later baseline assessment times. Despite this, assessment time remains an underutilized feature in other studies [11]. The role of assessment timing for predictive performance could also partially explain observed differences in predictive performance between test sets derived from EMSCI, which was also used for training, and the external validation data from the Sygen clinical trial.

A key contribution was our simulation framework to quantify the implications of using inevitably imperfect prediction models to generate synthetic controls as counterfactuals. Synthetic controls derived from the best prediction model had good agreement with the median of the ground truth in this simulation while displaying decreased variability compared to the random controls across a range of patient inclusion criteria and cohort sizes. Our subsequent analysis based on the NISCI trial shows that a single-arm trial enabled by synthetic controls is feasible. The primary outcome for placebo and synthetic controls does not show significant differences. This suggests that synthetic controls generated from ML models trained on a large observational dataset can provide a suitable counterfactual outcome in a single-arm trial under conditions similar to our study. A subsequent power analysis highlights the potential to either reduce the number of individuals recruited to detect a given treatment effect, or potentially detect smaller treatment effects if successfully recruiting the same number of participants.

Our study shows promise for using a synthetic control framework given small, heterogeneous cohorts to either reduce inter-cohort variability or to reduce the number of patients required. Synthetic controls could effectively support clinical trial designs that do not include a placebo group, such as concurrent control, baseline control, and external control trials [42]. However, this comes with caveats to consider: synthetic controls do not account for sample bias, placebo effects, changes in management and standard of care, or negative treatment administration effects for invasive treatments [42]. Moreover, whether synthetic controls and trial patient cohorts originate from comparable distributions, a key assumption, needs to be carefully accounted for. The NISCI trial underpinning our case study was conducted within the EMSCI network, suggesting better agreement between training data and the study population of interest than might be expected in other scenarios. However, a translation of models for clinical trials performed at other institutions warrants further investigation given possible differences in patient management and rehabilitation protocols. The use of biased synthetic controls due to changes in population distribution between the training data and the targeted single-arm cohort risks an increase in inferential errors (type I and II) depending on the nature of the distributional drift. Variability and uncertainty in predictions of recovery used as synthetic controls can also affect the conclusions potentially drawn for a clinical trial depending on the characteristics of the cohort studied, as the range of prediction errors differs between injury severities, for example. Accordingly, the validity of the synthetic control mechanism used should be established for the respective cohort studied as part of a particular clinical trial. Furthermore, potential bias in synthetic controls could increase the chance of either a false positive or a false negative finding if recovery is under- or overestimated, respectively. The results of our simulation study indicate that this scenario can occur for some prediction models, while the CNN used is not affected by this issue. Nonetheless, utilizing EMSCI, a multinational observational study continuously enrolling patients for more than 20 years, and data augmentation to quantify prediction uncertainty makes the best performing model from our study a valuable starting point for further exploration in other environments. Nonetheless, any future application of synthetic controls would have to ascertain the suitability of the synthetic controls as counterfactuals based on data specific to the trial in question or the institutional environment in which the trial is conducted. It should be stressed that the adaptation of a synthetic control framework to trials conducted outside large established networks such as EMSCI would likely require additional validation and adaptation. If an existing, pre-trained model such as the CNN developed as part of this study were to be used, a comprehensive comparison between the cohort used for training this model and historic data collected at participating trial sites could establish whether any differences in the distribution of patient characteristics and/or recovery patterns exist. If such differences were observed, the model used for deriving synthetic controls could be fine-tuned using the historical data from the relevant cohort. Such a two-step process would allow the use of the comprehensive data provided by large observational studies such as EMSCI [43] or the North American Clinical Trials Network [44], while ensuring that synthetic controls reflect the cohort studied as well as possible. The use of a hybrid design which incorporates a large treatment group, a smaller control group, and synthetic controls could enable further validation of the synthetic control mechanism by allowing assessment of whether synthetic controls are in sufficient agreement with the observed recovery in the control group, similar to the retrospective analysis of the NISCI trial presented in our case study. The comparisons possible by using such a hybrid design would further allow assessment of whether placebo or negative effects of invasive treatment administration potentially occurred, as these could be reflected in positive or negative deviations in recovery between the concurrent and synthetic control groups. While the synthetic control mechanism could not necessarily be adjusted during the analysis of a particular trial to reflect such deviations, the possibility to detect such differences would be valuable for future trial planning. The use of a hybrid design could further help alleviate ethical concerns arising from premature termination of clinical trials [45] because of the smaller number of individuals required and could provide additional insights, for example, by enabling the detection of deviations of a synthetic control mechanism from observed recovery patterns. Considering these challenges, the synthetic control framework is proposed for initial use in phase II trials, which aim to establish efficacy in smaller patient groups before advancing a treatment for final confirmation of safety and efficacy in randomized phase III trials. Given the challenges surrounding trials for rare conditions like SCI, the potential risks associated with synthetic controls might be acceptably balanced by an improved ability to plan and complete trials with small heterogeneous populations.

Additionally, we focused our counterfactual analysis solely on segmental ISNCSCI scores and demographics as input variables. Additional data modalities, such as imaging [46, 47] and omics [48, 49], would likely enhance the understanding of recovery trajectories and improve their prediction. These variables could also potentially represent unmeasured confounders, which would have to be accounted for in a single-arm trial. Including these additional modalities, and potentially information on secondary complications occurring or medications provided, could provide further assurance that relevant confounders are accounted for. However, currently, no large databases systematically collecting these data types exist. The ISNCSCI assessment provides the best balance between data availability and detailed information necessary to justify its application for generating synthetic controls. While the ISNCSCI assessment versions in Sygen and EMSCI differ, the large number of patients available in Sygen makes it the best available source for external validation. Future research should delve into integrating additional data types, once they become accessible, and further validation using external data. Moreover, an in-depth consideration of prediction uncertainties would be desirable. While we incorporate a first notion of assessment uncertainties, more comprehensive estimates as a function of myotome or dermatome and time remain missing. We acknowledge that trial design relying solely on synthetic controls presents potential limitations with respect to the validation of these controls. As such, it might be necessary to consider hybrid designs, which aim to maximize the number of individuals receiving an investigative treatment while still recruiting a small group of concurrent controls to monitor the validity of synthetic controls.

## Conclusions

In summary, this study offers a first proof of principle to motivate the use of synthetic controls for clinical trials in SCI. We conducted a comprehensive evaluation to identify the most suitable prediction framework for this objective and demonstrated that predictions of segmental motor recovery based on deep learning frameworks are suitable to derive synthetic controls. Based on a retrospective analysis of a recent clinical trial in SCI, our results highlight that alternative trial designs are feasible and could result in smaller cohort sizes while maintaining the ability to detect commonly targeted treatment effect sizes.

## Supplementary Information

Additional file 1: Sections 1–2. Section 1 — Supplementary Methods. Section 2 — Supplementary Results. Figures S1-S6. Figure S1 — Schematic overview of the deep learning architectures. Figure S2 — Consort diagram for EMSCI cohort. Figure S3 — Consort diagram for Sygen cohort. Figure S4 — RMSE_bl.NLI_ as function of time of initial assessment. Figure S5 — RMSE_bl.NLI_ as function of time of initial assessment for EMSCI cohort subset according to NISCI inclusion criteria. Figure S6 — Importance ranking of interpretability SHAP scores. Tables S1-S15. Table S1 — Hyperparameters for tree-based models. Table S2 — Hyperparameters for deep learning models. Table S3 — Characteristics of EMSCI and Sygen cohorts used for machine learning benchmark in comparison with subsets excluded. Table S4 — Number of instances in EMSCI with missing age. Table S5 — Number of instances in EMSCI within each AIS grade with imputed VAC. Table S6 — Number of instances in EMSCI within each AIS grade with imputed DAP. Table S7 — Results of the model benchmark. Table S8 — Performance on the EMSCI dataset stratified by AIS grade. Table S9 — Performance on the Sygen dataset stratified by AIS grade. Table S10 — Median of mean residuals below the NLI. Table S11 — Median of mean residual below NLI on the EMSCI dataset stratified by AIS grade. Table S12 — Median of mean residual below NLI on the Sygen dataset stratified by AIS grade. Table S13 — Benchmark on EMSCI cohort with NISCI inclusion criteria. Table S14 — Benchmark of CNN multi-modal trained on the same data as before and all combinations of time points. Table S15 — Distribution of group-level differences in mean LEMS_impr_.

## Data Availability

Anonymized data used in this study will be made available upon request to the corresponding author and in compliance with the General Data Protection Regulation (EU GDPR). We publish all code required to reproduce the presented results in our GitHub repository: https://gitlab.ethz.ch/BMDSlab/SCI/sci-in-silico-trials.

## Abbreviations

AIS: American Spinal Injury Association Impairment Scale
ASIA: American Spinal Injury Association
CNN: Convolutional neural network
Control group: Group established to evaluate randomized and synthetic control mechanisms in repeated simulations. Individuals in this group would not receive investigative treatment in RCT and would reflect natural recovery for comparison with the treatment group
DAI: Days after injury
DAP: Deep anal pressure
EMA: European Medicines Agency
EMSCI: European Multicenter Study about Spinal Cord Injury
GNN: Graph neural network
ISNCSCI: International Standards for Neurological Classification of Spinal Cord Injury
LEMS: Lower extremity motor score
LEMS_impr._: Improvement in lower extremity motor score between baseline assessment and follow-up assessment; computed as LEMS_follow__-up_– LEMS_baseline_
ΔLEMS_impr__._: Difference in lower extremity motor score improvements between two groups (e.g., control group and zero treatment group)
LTS: Light touch score
ML: Machine learning
MS: Motor score
NISCI: Nogo Inhibition in spinal cord injury
NLI: Neurological level of injury
PPS: Pin prick score
RCT: Randomized clinical trial
RMSE: Root mean squared error
RMSE_bl.NLI_: Root mean squared error below the initial neurological level of injury
SCI: Spinal cord injury
SHAP: SHapley Additive exPlanations
UEMS: Upper extremity motor score
VAC: Voluntary anal contraction
Zero treatment group: Group established to evaluate randomized and synthetic control mechanisms in repeated simulations. Individuals in this group would receive investigative treatment in RCT but reflect natural recovery in this study. Hence, a treatment effect of zero is expected in comparison with either control mechanism

## References

[1] Tator CH. Update on the pathophysiology and pathology of acute spinal cord injury. Brain Pathol. 1995;5:407–13. 8974623 10.1111/j.1750-3639.1995.tb00619.x

[2] Badhiwala JH, Wilson JR, Fehlings MG. Global burden of traumatic brain and spinal cord injury. Lancet Neurol. 2019;18:24–5. 30497967 10.1016/S1474-4422(18)30444-7

[3] Post M, Noreau L. Quality of life after spinal cord injury. J Neurol Phys Ther. 2005;29:139–46. 16398946 10.1097/01.npt.0000282246.08288.67

[4] Mulcahey MJ, Jones LAT, Rockhold F, Rupp R, Kramer JLK, Kirshblum S, et al. Adaptive trial designs for spinal cord injury clinical trials directed to the central nervous system. Spinal Cord. 2020;58:1235–48. 32939028 10.1038/s41393-020-00547-8

[5] Stephenson D, Ollivier C, Brinton R, Barrett J. Can innovative trial designs in orphan diseases drive advancement of treatments for common neurological diseases? Clin Pharmacol Ther. 2022;111:799–806. 35034352 10.1002/cpt.2528 PMC9305159

[6] Weidner N, Abel R, Maier D, Röhl K, Röhrich F, Baumberger M, et al. Safety and efficacy of intrathecal antibodies to Nogo-A in patients with acute cervical spinal cord injury: a randomised, double-blind, multicentre, placebo-controlled, phase 2b trial. Lancet Neurol. 2025;24:42–53. 39706632 10.1016/S1474-4422(24)00447-2

[7] Thorlund K, Dron L, Park JJH, Mills EJ. Synthetic and external controls in clinical trials– a primer for researchers. Clin Epidemiol. 2020;12:457–67. 32440224 10.2147/CLEP.S242097 PMC7218288

[8] Jordan MI, Mitchell TM. Machine learning: trends, perspectives, and prospects. Science. 2015;349:255–60. 26185243 10.1126/science.aaa8415

[9] Azizi Z, Zheng C, Mosquera L, Pilote L, El Emam K, GOING-FWD Collaborators. Can synthetic data be a proxy for real clinical trial data? A validation study. BMJ Open. 2021;11:e043497. 33863713 10.1136/bmjopen-2020-043497 PMC8055130

[10] Chen T, Chen H. Universal approximation to nonlinear operators by neural networks with arbitrary activation functions and its application to dynamical systems. IEEE Trans Neural Netw. 1995;6:911–7. 18263379 10.1109/72.392253

[11] Håkansson S, Tuci M, Bolliger M, Curt A, Jutzeler CR, Brüningk S. Data-driven prediction of spinal cord injury recovery: an exploration of current status and future perspectives. medRxiv. 2024;:2024.05.03.24306807. 10.1016/j.expneurol.2024.114913 39097073

[12] Wilson JR, Cadotte DW, Fehlings MG. Clinical predictors of neurological outcome, functional status, and survival after traumatic spinal cord injury: a systematic review. J Neurosurg Spine. 2012;17(1 Suppl):11–26. 22985366 10.3171/2012.4.AOSPINE1245

[13] Balbinot G, Li G, Kalsi-Ryan S, Abel R, Maier D, Kalke Y-B, et al. Segmental motor recovery after cervical spinal cord injury relates to density and integrity of corticospinal tract projections. Nat Commun. 2023;14:723. 36759606 10.1038/s41467-023-36390-7 PMC9911610

[14] Brüningk SC, Bourguignon L, Lukas LP, EMSCI Study Group, Maier D, Abel R, et al. Prediction of segmental motor outcomes in traumatic spinal cord injury: advances beyond sum scores. Exp Neurol. 2024;380:114905. 39097076 10.1016/j.expneurol.2024.114905

[15] Buri M, Tanadini LG, Hothorn T, Curt A. Unbiased recursive partitioning enables robust and reliable outcome prediction in acute spinal cord injury. J Neurotrauma. 2022;39:266–76. 33619988 10.1089/neu.2020.7407

[16] Single-arm trials as pivotal evidence for the authorisation of medicines in the EU. https://www.ema.europa.eu/en/news/single-arm-trials-pivotal-evidence-authorisation-medicines-eu. Accessed 17 May 2024.

[17] Rupp R, Biering-Sørensen F, Burns SP, Graves DE, Guest J, Jones L, et al. International standards for neurological classification of spinal cord injury: revised 2019. Top Spinal Cord Inj Rehabil. 2021;27:1–22. 34108832 10.46292/sci2702-1 PMC8152171

[18] Kirshblum SC, Biering-Sorensen F, Betz R, Burns S, Donovan W, Graves DE, et al. International standards for neurological classification of spinal cord injury: cases with classification challenges. J Spinal Cord Med. 2014;37:120–7. 24559416 10.1179/2045772314Y.0000000196 PMC4066420

[19] Geisler FH, Coleman WP, Grieco G, Poonian D, Sygen Study Group. The Sygen multicenter acute spinal cord injury study. Spine. 2001;26(24) Suppl:S87–98. 10.1097/00007632-200112151-00015 11805614

[20] Steeves JD, Lammertse D, Curt A, Fawcett JW, Tuszynski MH, Ditunno JF, et al. Guidelines for the conduct of clinical trials for spinal cord injury (SCI) as developed by the ICCP panel: clinical trial outcome measures. Spinal Cord. 2007;45:206–21. 17179972 10.1038/sj.sc.3102008

[21] Marino RJ, Burns S, Graves DE, Leiby BE, Kirshblum S, Lammertse DP. Upper- and lower-extremity motor recovery after traumatic cervical spinal cord injury: an update from the national spinal cord injury database. Arch Phys Med Rehabil. 2011;92:369–75. 21353821 10.1016/j.apmr.2010.09.027

[22] Pedregosa F, Varoquaux G, Gramfort A, Michel V, Thirion B, Grisel O, et al. Scikit-learn: machine learning in Python. J Mach Learn Res. 2011;abs/1201.0490:2825–30.

[23] Lundberg SM, Lee S-I. A unified approach to interpreting model predictions. In: Proceedings of the 31st International Conference on Neural Information Processing Systems. Red Hook: Curran Associates Inc.; 2017. p. 4768–77.

[24] Hoerl AE, Kennard RW. Ridge regression: applications to nonorthogonal problems. Technometrics. 2012;12(1):69–82.

[25] Zou H, Hastie T. Regularization and variable selection via the elastic net. J R Stat Soc Ser B Stat Methodol. 2005;67:301–20.

[26] Breiman L. Random forests. Mach Learn. 2001;45:5–32.

[27] Chen T, Guestrin C. XGBoost: a scalable tree boosting system. In: Proceedings of the 22nd ACM SIGKDD International Conference on Knowledge Discovery and Data Mining. Association for Computing Machinery. p. 785–94.

[28] Li L, Jamieson K, DeSalvo G, Rostamizadeh A, Talwalkar A. Hyperband: a novel bandit-based approach to hyperparameter optimization. J Mach Learn Res. 2018;18:1–52.

[29] Abadi M, Agarwal A, Barham P, Brevdo E, Chen Z, Citro C, et al. TensorFlow: large-scale machine learning on heterogeneous distributed systems. 2016.

[30] Fey M, Lenssen JE. Fast graph representation learning with PyTorch geometric. arXiv [cs.LG]. 2019.

[31] Bye E, Glinsky J, Yeomans J, Hungerford A, Patterson H, Chen L, et al. The inter-rater reliability of the 13-point manual muscle test in people with spinal cord injury. Physiother Theory Pract. 2019. 10.1080/09593985.2019.1685033. 31674263 10.1080/09593985.2019.1685033

[32] Dietz VA, Roberts N, Knox K, Moore S, Pitonak M, Barr C, et al. Fighting for recovery on multiple fronts: the past, present, and future of clinical trials for spinal cord injury. Front Cell Neurosci. 2022;16:977679. 36212690 10.3389/fncel.2022.977679 PMC9533868

[33] Popat S, Liu SV, Scheuer N, Hsu GG, Lockhart A, Ramagopalan SV, et al. Addressing challenges with real-world synthetic control arms to demonstrate the comparative effectiveness of Pralsetinib in non-small cell lung cancer. Nat Commun. 2022;13:3500. 35715405 10.1038/s41467-022-30908-1 PMC9205915

[34] Graves DE, Frankiewicz RG, Donovan WH. Construct validity and dimensional structure of the ASIA motor scale. J Spinal Cord Med. 2006;29:39–45. 16572564 10.1080/10790268.2006.11753855 PMC1864793

[35] Snider BA, Eren F, Reeves RK, Rupp R, Kirshblum SC. The international standards for neurological classification of spinal cord injury: classification accuracy and challenges. Top Spinal Cord Inj Rehabil. 2023;29:1–15. 36819931 10.46292/sci22-00036 PMC9936898

[36] Harrigan CF, Morgenshtern G, Goldenberg A, Chevalier F. Considerations for visualizing uncertainty in clinical machine learning models. arXiv [cs.HC]. 2022.

[37] Kapoor D, Xu C. Spinal cord injury AIS predictions using machine learning. eNeuro. 2023;10. 10.1523/ENEURO.0149-22.2022. 10.1523/ENEURO.0149-22.2022 PMC9831144 36543536

[38] Beninato M, O’Kane KS, Sullivan PE. Relationship between motor FIM and muscle strength in lower cervical-level spinal cord injuries. Spinal Cord. 2004;42:533–40. 15224086 10.1038/sj.sc.3101635

[39] Saghazadeh A, Rezaei N. The role of timing in the treatment of spinal cord injury. Biomed Pharmacother. 2017;92:128–39. 28535416 10.1016/j.biopha.2017.05.048

[40] Velstra I-M, Bolliger M, Krebs J, Rietman JS, Curt A. Predictive value of upper limb muscles and grasp patterns on functional outcome in cervical spinal cord injury. Neurorehabil Neural Repair. 2016;30:295–306. 26156192 10.1177/1545968315593806

[41] Scheel-Sailer A, Sailer CO, Lampart P, Baumberger M, Berger M, Mueller G, et al. Examinations and assessments in patients with a newly acquired spinal cord injury– retrospective chart analysis as part of a quality improvement project. Swiss Med Wkly. 2020;150:w20291. 32730632 10.4414/smw.2020.20291

[42] Lammertse D, Tuszynski MH, Steeves JD, Curt A, Fawcett JW, Rask C, et al. Guidelines for the conduct of clinical trials for spinal cord injury as developed by the ICCP panel: clinical trial design. Spinal Cord. 2007;45:232–42. 17179970 10.1038/sj.sc.3102010 PMC4106695

[43] Bourguignon L, Tong B, Geisler F, Schubert M, Röhrich F, Saur M, et al. International surveillance study in acute spinal cord injury confirms viability of multinational clinical trials. BMC Med. 2022;20:225. 35705947 10.1186/s12916-022-02395-0 PMC9202190

[44] Fehlings MG, Boakye M, Ditunno JF Jr, Vaccaro AR, Rossignol S, Burns AS, editors. North American clinical trials network: building a clinical trials network for spinal cord injury. In: Essentials of spinal cord injury. Stuttgart: Georg Thieme Verlag; 2013.

[45] Curt A, Levi AD, Schwab JM. Challenges to translation and the hippocratic oath by premature termination of spinal cord stem cell-based trials. JAMA Neurol. 2017;74:635–6. 28437542 10.1001/jamaneurol.2017.0318

[46] Smith AC, Albin SR, O’Dell DR, Berliner JC, Dungan D, Sevigny M, et al. Axial MRI biomarkers of spinal cord damage to predict future walking and motor function: a retrospective study. Spinal Cord. 2021;59:693–9. 33024298 10.1038/s41393-020-00561-w PMC8021607

[47] Matsushita A, Maeda T, Mori E, Yuge I, Kawano O, Ueta T, et al. Can the acute magnetic resonance imaging features reflect neurologic prognosis in patients with cervical spinal cord injury? Spine J. 2017;17:1319–24. 28501580 10.1016/j.spinee.2017.05.009

[48] Harrington GMB, Cool P, Hulme C, Osman A, Chowdhury JR, Kumar N, et al. Routinely measured hematological markers can help to predict American Spinal Injury Association impairment scale scores after spinal cord injury. J Neurotrauma. 2021;38:301–8. 32703074 10.1089/neu.2020.7144 PMC7826437

[49] Skinnider MA, Rogalski J, Tigchelaar S, Manouchehri N, Prudova A, Jackson AM, et al. Proteomic portraits reveal evolutionarily conserved and divergent responses to spinal cord injury. Mol Cell Proteomics. 2021;20:100096. 34129941 10.1016/j.mcpro.2021.100096 PMC8260874

