# Supplementary material for: Exploring synthetic controls in rare diseases with a proof of concept in spinal cord injury
Source: BMC Med. 2025 Oct 24;23:581. doi: 10.1186/s12916-025-04405-3 (PMC12551237; doi:10.1186/s12916-025-04405-3)
Supplement: Supplementary file 1 — Additional file 1: Sections 1–2. Section 1 — Supplementary Methods. Section 2 — Supplementary Results. Figures S1-S6. Figure S1 — Schematic overview of the deep learning architectures. Figure S2 — Consort diagram for EMSCI cohort. Figure S3 — Consort diagram for Sygen cohort. Figure S4 — RMSEbl.NLI as function of time of initial assessment. Figure S5 — RMSEbl.NLI as function of time of initial assessment for EMSCI cohort subset according to NISCI inclusion criteria. Figure S6 — Importance ranking of interpretability SHAP scores. Tables S1-S15. Table S1 — Hyperparameters for tree-based models. Table S2 — Hyperparameters for deep learning models. Table S3 — Characteristics of EMSCI and Sygen cohorts used for machine learning benchmark in comparison with subsets excluded. Table S4 — Number of instances in EMSCI with missing age. Table S5 — Number of instances in EMSCI within each AIS grade with imputed VAC. Table S6 — Number of instances in EMSCI within each AIS grade with imputed DAP. Table S7 — Results of the model benchmark. Table S8 — Performance on the EMSCI dataset stratified by AIS grade. Table S9 — Performance on the Sygen dataset stratified by AIS grade. Table S10 — Median of mean residuals below the NLI. Table S11 — Median of mean residual below NLI on the EMSCI dataset stratified by AIS grade. Table S12 — Median of mean residual below NLI on the Sygen dataset stratified by AIS grade. Table S13 — Benchmark on EMSCI cohort with NISCI inclusion criteria. Table S14 — Benchmark of CNN multi-modal trained on the same data as before and all combinations of time points. Table S15 — Distribution of group-level differences in mean LEMSimpr. [file 12916_2025_4405_MOESM1_ESM.pdf]

Additional File 1 for **Exploring synthetic controls in rare diseases with a proof of concept in spinal cord injury**

Louis P. Lukas, MSc[1,2,#], Samuel Håkansson, PhD[1,2,#], Miklovana Tuci, MSc[1,2,3], Abel Torres Espin, Prof[4,5,6], Rüdiger Rupp, Prof[7,8], Olga Taran, PhD[1,2], Norbert Weidner, Prof[7,8], Fred Geisler, PhD[9], Martin Schubert, PhD[3], Frank Röhrich, PhD[10], Yorck B. Kalke, PhD[11], Rainer Abel, Prof[12], Doris Maier, PhD[13], Harvinder S. Chhabra, MS[14], Thomas Liebscher, PhD[15], EMSCI study group[3], John L.K. Kramer, PhD[16,17,18,19], Marc Bolliger, PhD[3], Armin Curt, Prof[3], Catherine R. Jutzeler, Prof[1,2,*,†], Sarah C. Brüningk, Prof[1,20,*,†]

[1]Department of Health Sciences and Technology (D-HEST), ETH Zurich, Zürich, Switzerland
[2]SIB Swiss Institute of Bioinformatics, Lausanne, Switzerland
[3]Spinal Cord Injury Center, University Hospital Balgrist, University of Zurich, Zürich, Switzerland
[4]School of Public Health Sciences, University of Waterloo, Waterloo, Canada
[5]Department of Neurological Surgery, University of California San Francisco, San Francisco, USA
[6]Department of Physical Therapy, University of Alberta, Alberta, Canada
[7]Faculty for Medicine, Heidelberg University, Heidelberg, Germany
[8]Spinal Cord Injury Center, Heidelberg University Hospital, Heidelberg, Germany
[9]University of Saskatchewan, Saskatoon, Canada
[10]Spinal Cord Injury Center, Orthopaedic Department, Berufsgenossenschaftliches Klinikum Bergmannstrost Halle gGmbH, Halle, Germany
[11]Spinal Cord Injury Center Orthopaedic Department, Ulm University, Ulm, Germany
[12]Spinal Cord Injury Center, Bayreuth, Germany
[13]Spinal Cord Injury Center, Trauma Center Murnau, Murnau, Germany
[14]Department of Spine and Rehabilitation, Sri Balaji Action Medical Institute, New Delhi, India
[15]Treatment Centre for Spinal Cord Injuries, Trauma Hospital Berlin, Berlin, Germany
[16]International Collaboration on Repair Discoveries (ICORD), University of British Columbia, Vancouver, Canada
[17]Djavad Mowafaghian Centre for Brain Health, University of British Columbia, Vancouver, Canada
[18]Department of Anesthesiology, Pharmacology, and Therapeutics, Faculty of Medicine, University of British Columbia, Vancouver, Canada
[19]Hugill Centre for Anesthesia, University of British Columbia, Vancouver, Canada
[20] Department of Radiation Oncology, Inselspital, Bern University Hospital and University of Bern, Switzerland

[#]Equal contributors
[†]Equal contributors
[*]Corresponding authors:
Sarah C. Brüningk: sarah.brueningk@hest.ethz.ch
Catherine R. Jutzeler: catherine.jutzeler@hest.ethz.ch

List of abbreviations

AIS	American Spinal Injury Association Impairment Scale
ASIA	American Spinal Injury Association
CNN	convolutional neural network
control group	Group established to evaluate randomized and synthetic control mechanisms in repeated simulations. Individuals in this group would not receive investigative treatment in RCT and reflect natural recovery for comparison with the treatment group.
DAI	days after injury
DAP	deep anal pressure
EMA	European Medicines Agency
EMSCI	European Multicenter Study about Spinal Cord Injury
GNN	graph neural network
ISNCSCI	International Standards for Neurological Classification of Spinal Cord Injury
LEMS	lower extremity motor score
$LEMS_{impr.}$	improvement in lower extremity motor score between baseline assessment and follow-up assessment; computed as $LEMS_{follow\text{-}up}$ - $LEMS_{baseline}$
$\Delta LEMS_{impr.}$	difference in lower extremity motor score improvements between two groups (e.g. control group and zero treatment group)
LTS	light touch score
ML	machine learning
MS	motor score
NISCI	Nogo Inhibition in spinal cord injury
NLI	neurological level of injury
PPS	pin prick score

RCT	randomized clinical trial
RMSE	root mean squared error
$RMSE_{bl.NLI}$	root mean squared error below the initial neurological level of injury
SCI	spinal cord injury
SHAP	SHapley Additive exPlanations
UEMS	upper extremity motor score
VAC	voluntary anal contraction
zero treatment group	Group established to evaluate randomized and synthetic control mechanisms in repeated simulations. Individuals in this group would receive investigative treatment in RCT but reflect natural recovery in this study. Hence, a treatment effect of zero is expected in comparison with either control mechanism.

1. Supplementary Methods

1.1 Input data

The European Multicenter Study about Spinal Cord Injury (EMSCI) is an observational study collecting data in 32 centers across Europe and one in India. Data for participating patients is collected at five time points in the first year after injury: very acute (0 to 15 days after injury [DAI]), acute I (16 to 40 DAI), acute II (70 to 98 DAI), acute III (150 to 186 DAI), chronic (300 to 546 DAI). Sygen assessed patients at five time points in the first year (weeks 1, 4, 8, 26, 52 after injury).

The ISNCSCI exam provides an assessment of body structure and function by scoring key muscle function at ten myotomes on both sides of the body on a range from 0 (full paralysis) to 5 (active movement against full resistance). Additionally sensory information is assessed based on light touch and pinprick sensation on a range from 0 (no sensation) to 2 (normal sensation).

1.2 Data preprocessing

Missing values for age at time of injury were imputed using mean age. Binary values for voluntary anal contraction (VAC) and deep anal pressure (DAP) were imputed using the mode within each AIS grade (VAC: A, B, C - No, D - Yes; DAP; A - No, B, C, D - Yes). Note that missing values were not imputed for motor and sensory scores, AIS grade, NLI, and timings of initial and endpoint assessment as these variables were deemed more sensitive to imputation in regard to the recovery process. If any of the missing variables were not present in the data for a patient, the patient was not included. Motor, light touch, and pinprick scores were scaled using the MinMax scaler (scikit-learn). AIS grade (one-hot encoded, first column dropped, leaving columns AIS B, C, and D), sex, VAC, and DAP were encoded as single binary variables. Age, NLI, time of initial assessment and time of outcome assessment were scaled with StandardScaler (scikit-learn).

1.3 Model benchmark

1.3.1 Model architectures evaluated

Baseline models: We used regularized linear regression (ridge regression [24] and elastic net [25]) and tree-based architectures (random forest [RF] [26], extreme gradient boosting [XGBoost] [27]). Each model predicts motor scores for a single spinal segment at a time, resulting in ten models (each model predicts left and right motor score). Moreover, separate model instances were trained for motor scores equal to zero, or above leading to twenty model instances being trained independently for each architecture to predict all motor scores (see **Tables 2** and **S7** for comparison of performance). The rationale behind the separation of models between score 0 and >0 is because the MS scale is ordinal, where patients that start at score 0 tend to stay at 0. Right and left side indicators were provided as a binary input feature. For each patient, only the myotomes below the level of injury were included in the training data for the baseline models. The output was clipped after prediction to the range of a motor score, [0, 5]. Baseline models were implemented using the *scikit-learn* [22] library in Python using negative root mean square error as the loss function. Grid search was performed to optimize model hyperparameters (see **Table S1** for details).

Convolutional and transformer neural networks: CNNs are widely used for image and (time-)series processing but can be utilized in similar tasks where the data can be modeled as one dimensional sequences or image-like objects. Three versions of CNNs were created as outlined in **Figure S1 A-C**: (1) *multi-modal*, (2) *(multi-modal) ensemble, and* (3) *block (input).* In *multi-modal CNN*, the input data is divided into three parts - MS, LTS, and PPS, and tabular data. LTS and PPS are added on top of each other as a new dimension, in the same way as a layer of green color is layered upon red in an RGB image. The MS and LTS/PPS inputs are processed in 1D convolutions and 2D convolutions, respectively, while the tabular data is processed with fully connected layers. The (1D and 2D) convolutions and the (intermediate) output from the fully connected layer from the tabular data are then concatenated in latent space before predicting the output motor scores. The *(multi-modal) ensemble CNN* replaces the final fully connected layer with a weighted average decision by the three modalities. The *block (input) CNN* replaces the separate MS and LT/PP inputs with a (28,2,3)-dimensional input (myotomes, left/right, MS/LT/PP). Any motor scores that are not represented in ISNCSCI are imputed with 5 above the NLI, the score of L2 above L2 until NLI, and the value of S1 below S1. Two versions of transformers were modeled (**Figure S1 D-E**): *multi-modal* and *block (input).* In a similar fashion to the CNN models, the *multi-modal transformer* takes MS, LTS/PPS, and tabular data as three separate inputs. The *block transformer* uses the same (imputed) block input as the *block CNN* and uses 3D positional encoding (Supplementary Methods). Both versions utilize multi-head attention. The output function of the CNNs and transformers was clipped

to [0, 5]. Hyperparameterization (see **Table S2**) for the CNN and transformer models was performed using Hyperband [28].

Graph neural network (GNN): ISNCSCI examination results for key myotomes at early and late time points were represented as regular graphs, in which each node represents a myotome (**Figure S1 F**). MS, LTS, and PPS for myotomes C5-T1 and L2-S1 were used as node features. Additional features were processed using fully connected layers. Latent representations resulting from the GNN and fully connected layers were concatenated and used for the final prediction. GNNs were trained on a node-level regression task, in which motor scores for all myotomes (nodes) were predicted simultaneously. Hyperparameters (see **Table S2**) were optimized using grid search.

The ML model layers are connected to reflect how myotomes (muscle segments controlled by individual spinal nerves) interact. For instance, the models connect data from the left and right C5 motor scores to their respective C6 segments, where C5 left directly connects to C6 left, but not to C6 right. These ML architectures differ in their efficiency for different problem formulations which can be exemplified by their initial design purposes. For example, CNNs are optimized for image recognition, Transformers excel in processing text, and GNNs are effective for analyzing structures like network topologies and molecular formations. The choice of architecture often depends on how a problem is conceptualized; for instance, transforming a graph into an image allows for different computational approaches, which can be crucial in developing models for predicting recovery outcomes in medical settings. An overview of the differences between the architectures is visualized in **Figure S1**.

All deep learning models used the root mean squared error below NLI (as assessed at the earliest available time point) as the loss function. The deep learning models used only one model instance to output all 20 motor scores. Five-fold cross-validation was used to evaluate the models, where the cohort is divided into five subcohorts; four for the training set (80% of the data) and one for the test set (20% of the data). This process is repeated five times, such that each subcohort is used for evaluation once (see **Figure 1**).

In the model benchmark, the best model version of the CNN and transformers was used: *CNN* is the *multi-modal CNN*, and *transformer* is the *multi-modal transformer.*

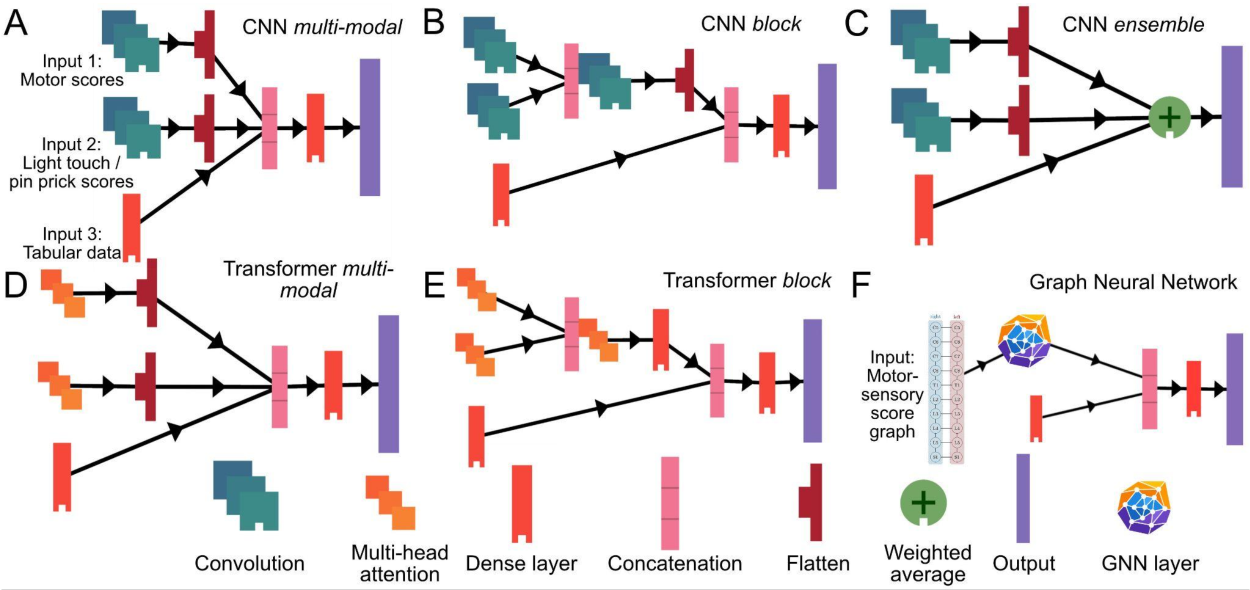

Supplementary Figure S1. Schematic overview of the deep learning architectures. Input 1 is motor scores of size 10x2, input 2 is a concatenation of light touch and pinprick scores with size 28x2x2, and the tabular data is of size 10x1 and contains AIS grade, NLI, VAC, DAP, age, and sex. The output is of size 20x1 and represents the 20 motor scores. In the CNN *block* (B) and Transformer *block* (E), the two convolutions and the two multi-head attention architectures that are concatenated into one represent the process of concatenating and imputing the MS, LTS, and PPS to one *block*. For further details about hyperparameters and optimized parameters, see **Table S2.**

1.3.2 Hyperparameters evaluated

Hyperparameters of the regression and tree-based models were tuned using GridSearch with internal five-fold cross-validation. Hyperparameters evaluated for tree-based architectures are shown in supplementary **Table S1**. Hyperparameters evaluated for elastic net regression include alpha (0.0001, 0.01) and the L1-ratio (1e-7, 2.15e-5. 4.64e-3, 1), while for ridge regression alpha (0, 0.01, 0.0001) was optimized. Hyperparameters of deep learning models were evaluated according to information in Supplementary **Table S2**.

Supplementary Table S1: Hyperparameters for tree-based models.

	**Max depth**	**Number of estimators**	**Min samples split**	**Max number features at split**	**Subsample ratio of features per tree**	**Min samples leaf**	**Bootstrap**	**Subsample**	**Eta / boosting learning rate**	**Loss function**
XGB	3, 5, 10	500	N/A	N/A	0.5, 0.75, 1	N/A	N/A	1	0.001, 0.01, 0.1	negative RMSE
Random forest	3, 5, 7, None	50, 200, 1000	3, 5, 10	square root	N/A	3, 6, 9	True	N/A	N/A	N/A
Random forest (MS same)	5, 7, None	200, 1000	3, 5	square root	N/A	3, 6	True	N/A	N/A	N/A

Supplementary Table S2: Hyperparameters for deep learning models. Optimal hyperparameter values for CNN and transformer architectures were determined using Hyperbands (batch size: 32, 64, 128, 200, 256; iterations: 2; factor: 3; epochs: 2000, early stopping patience: 200). Optimal hyperparameter values for the GNN architecture were determined using grid search. Optimal parameters are marked with *.

	**Number of layers**	**Layer type**	**Number of heads**	**Head size**	**Feed-forward dimension**	**Number of filters**	**Kernel size**	**Embedding dimension**	**Pool size**	**Dropout rate**	**Batch normali-sation**	**L2 regulari-sation**	**Learning rate**	**Batch size**	**Trainable parameters**	**Non-trainable parameters**
CNN multimodal	1*, 2	N/A	N/A	N/A	N/A	4, 8, 12*, 16	3, 5, 7*	N/A	N/A	0.0, 0.1, 0.2*	N/A	0.0*, 0.001, 0.002	0.00001, 0.0001, 0.001*	32, 64*, 128, 200, 256	17580	48
CNN ensemble	1*, 2	N/A	N/A	N/A	N/A	4, 8, 12, 16*	3, 5, 7*	N/A	N/A	0.1, 0.2*, 0.3, 0.4, 0.5	N/A	0.0*, 0.001, 0.002	0.00001, 0.0001, 0.001*	32, 64*, 128, 200, 256	14015	64
CNN block	1*, 2	N/A	N/A	N/A	N/A	4*, 8, 12, 16	3, 5*, 7	N/A	2*, 3, 4	0.0, 0.1*, 0.2	N/A	0.0*, 0.001, 0.002	0.00001, 0.0001*, 0.001	32*, 64, 128, 200, 256	10316	264

transformer multimodal	1*, 2, 3	N/A	1, 2*, 3	64, 128, 256*, 512	64, 128*, 256	N/A	N/A	N/A	N/A	0.0, 0.1*, 0.2	N/A	0.0, 0.0001*, 0.001	0.01, 0.001, 0.0001*	32*, 64, 128, 200, 256	155676	0
transformer block	1*, 2, 3	N/A	4, 5*, 6, 7, 8	N/A	128, 192*, 256	N/A	N/A	32, 48*, 64	N/A	0.0, 0.1, 0.2*, 0.3	N/A	0.0, 0.001, 0.002, 0.003, 0.004*, 0.005, 0.006, 0.007, 0.008, 0.009, 0.01	0.00001, 0.0001*, 0.001	32, 64*, 128, 200, 256	68964	0
GNN	1, 2*, 3, 4	GAT, SAGE*	GAT: 1	N/A	10, 50, 100*	N/A	N/A	N/A	N/A	N/A	True, False*	N/A	0.0001*, 0.00001	250	20908	0

1.3.3 Libraries used

Standard machine learning models (XGBoost, random forest, ridge, and elastic net regression) were implemented using scikit-learn 1.3.1 [22]. All CNN and transformer architectures were implemented using Keras 2.13.1 [29]. Graph neural networks were implemented using PyTorch geometric 2.5.0 [30].

1.3.4 Shapley values for interpretability analysis

SHAP (SHapley Additive exPlanations) analysis was performed to evaluate the importance of the patient features (on each of the 20 motor score outputs). SHAP uses the model and the data, perturbs (sets of) the input data, and inspects if the output of the model changes to find the importance of the features. We used DeepExplainer from the *shap* library [23] to calculate the SHAP from all instances in the test set for all five cross-validation folds, meaning that the interpretability is interpolated between five models. A ranking of the importance of the motor and sensory scores based on the medial and lateral distance from the endpoint motor score ($MS_{endpoint}$) was performed using the rank for each $MS_{endpoint}$ (**Figure 2H**). The mean ranking over all of the distances from $MS_{endpoint}$ was used to calculate the final ranking list. For example, for the MS endpoint LMS L3, RMS L5 has a distance of 2, and between the upper and lower motor scores, the distance is truncated such that the value between T1 and L2 is 1. An example: if the $MS_{endpoint}$ is LMS C8, if the ranking of LMS T1 (which has a distance of 1) is 1, and for LMS L4, the ranking of LMS L5 (which also has a distance of 1) is 3, the mean ranking for distance 1 is 2. All distances for motor and sensory scores are then ranked based on the mean ranking.

1.3.5 Performance evaluation

We employed five-fold cross-validation (80% training/ 20% test) stratified by AIS grade and split at the patient level to avoid information leakage between the datasets. An augmented version of the dataset was created with 100-fold bootstrapping of the input data by drawing a sample for each MS from a distribution that approximates the uncertainty of the relevant MS assessment (as previously reported [14], distribution based on [31]). Note that the relevant probability density functions were approximated from a two-observer assessment in multiple patients on only two myotomes, and using a 13-point grade scale for MS. While this indeed is not perfect, we use this approximation as a first estimate to incorporate a notion of assessment uncertainty here.
Test-set predictions are reported as median values with 2.5 and 97.5 percentiles based on the resulting 100 individually predicted motor score sequences for each sample. Performance was compared on the internal test set and external validation set (Sygen) in terms of root mean squared errors (RMSE) between the true and predicted MS sequences below the NLI at the initial assessment point ($RMSE_{bl.NLI}$). $RMSE_{bl.NLI}$ is used because the scores above NLI are usually trivial to predict. Sygen results are reported relative to the mean over the five cross-validation models. Given the longitudinal character of our input data, we assessed the change of $RMSE_{bl.NLI}$ over the initial assessment time with a 7-day rolling average and 2.5 and 97.5 percentiles. The objective is to investigate whether predicting patient trajectories becomes easier and more reliable at later time points compared to earlier ones.

1.4 Case study

The evaluation of the potential of synthetic controls is based on two steps. The outcome of interest in both cases is the difference in upper extremity motor scores ($UEMS_{impr.}$). First, a comparison of synthetic controls for both the placebo and treatment groups of the NISCI trial with the observed recovery of the placebo group is completed to ensure synthetic controls do not systematically over- or underestimate recovery. For this purpose, we compare the $UEMS_{impr.}$ of the placebo group as observed with the estimated $UEMS_{impr.}$ derived from the synthetic controls for the placebo and treatment group using a one-sample and two-sample Wilcoxon rank-sum test respectively. Second, power calculations for both a single-arm clinical trial employing synthetic controls, and an RCT using treatment and placebo control groups are performed to establish if a difference in the number of patients, which is expected to be required for detecting a particular treatment effect, exists. To determine this number for an RCT, we perform a power calculation based on a two-sample t-test ($n = (\sigma_1^2 + \sigma_2^2)(z_{1-\alpha/2} + z_{1-\beta})^2 / |\mu_2 - \mu_1|^2$; σ, standard deviation of outcome; μ, mean of outcome; z, parameters for standard normal distribution). To determine an equivalent number for a single-arm trial, in which all individuals enrolled receive the treatment under investigation, employing synthetic controls as introduced in this work, we consider a paired t-test ($n = (z_{1-\alpha/2} + z_{1-\beta})^2\sigma_{adap}/ \varepsilon_t^2$; $\sigma_{adap}=\sigma^2 + u^2$ where σ, standard deviation of outcome; u, uncertainty of predictions of outcome as assessed by the standard deviation of prediction error; $\varepsilon_t = (\mu_O + t) - \mu_R$ where μ_O is the mean of the outcome of interest, t the hypothetical treatment effect, and μ_R the mean value of the reference outcome, derived from the synthetic controls). Power calculations in both cases are performed at significance level alpha=0.05, and power beta=0.8. We adapt the power calculation for the single-arm trial by using the predicted recovery of the synthetic controls as the reference value (μ_O) and account for prediction uncertainty by adding the squared standard deviation of prediction residuals to the squared standard deviation of the outcome. We consider the subset of patients from the EMSCI data, who meet the inclusion criteria of the NISCI trial, to compute all relevant parameters. We analyze the sensitivity of the results by repeating the analysis 500 times and subsampling the set of individuals from whom the parameter estimates relevant for the power calculations are derived.

2. Supplementary Results

2.1 Data preprocessing

2.1.1 Cohort selection

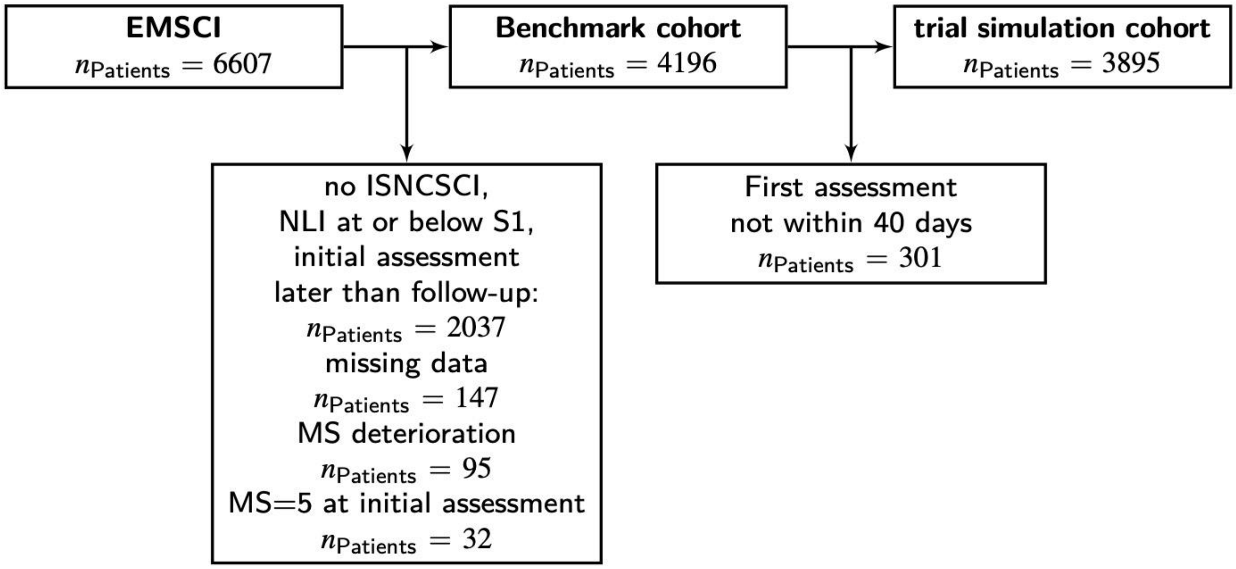

Supplementary Figure S2: Consort diagram of the benchmark and trial simulation cohort for EMSCI.

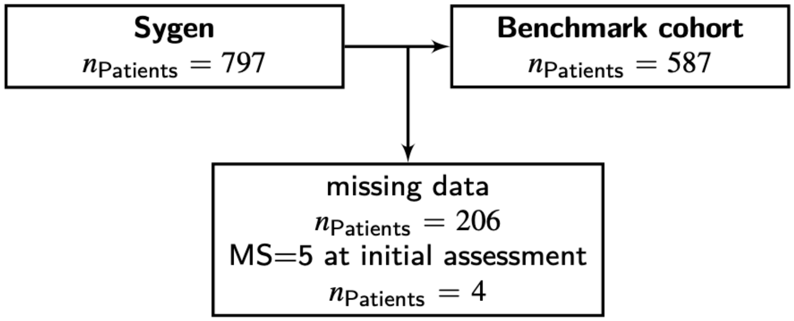

Supplementary Figure S3: Consort diagram of the benchmark cohort for Sygen.

Table S3.A and S3.B: Characteristics of EMSCI and Sygen cohorts used for machine learning benchmark in comparison with subsets excluded. Values reported at patient level and for earliest initial and output assessment time points for cohorts analyzed; cohort analyzed for EMSCI includes imputed values (see **Tables S4-6**). Statistically significant differences between included and excluded marked with *; continuous features compared using Wilcoxon-Rank-Sum test; categorical features compared using Chi-Squared test; alpha=0.003125 (adjusted for multiple testing using Bonferroni correction); distributions compared using data available/omitting missing data.

S3A		EMSCI (n=6607)		
		**benchmark** (n=4196)	**Excluded** (n=2411)	**p-value**
**Age [years]**, mean (SD)		46 (18)	51 (20)	≤0.0001* (4e-19)
	missing (n)	-	625	
**Time to first assessment (days)**, mean (SD)		22 (20)*	17 (11)*	≤0.0001* (4e-7)
	missing (n)	-	626	
**Time to first follow-up (days)**, mean (SD)		216 (91)*	304 (110)*	≤0.0001* (3e-14)
	missing (n)	-	2306	
**Sex (female)**, n (%)		928 (22.1)*	467 (19.4)*	0.0008*
	missing (n)	-	625	
**VAC (yes)**, n (%)		1280 (30.5)*	764 (31.7)*	≤0.0001* (7e-20)
	missing (n)	-	625	
**DAP (yes)**, n (%)		2197 (52.4)*	1126 (46.7)*	≤0.0001* (3e-14)
	missing (n)	-	625	
**Neurological level of injury**, n (%)				
	cervical	2256 (53.8)*	950 (39.4)*	0.001*
	thoracic	1563 (37.3)*	634 (26.3)*	
	lumbar	377 (9.0)*	198 (8.2)*	
	sacral	-*	4 (0.02)*	
	missing	-	625 (26.0)	
**Injury severity (AIS grade)**, n (%)				
	AIS A	1807 (43.1)*	553 (22.9)*	≤0.0001* (1e-23)
	AIS B	539 (12.5)*	197 (8.2)*	
	AIS C	761 (18.1)*	368 (15.3)*	
	AIS D	1089 (26.0)*	668 (27.7)*	
	missing	-	625 (26.0)	

S3B		Sygen (n=797)		
		**benchmark** (n=587)	**Excluded** (n=210)	**p-value**
**Age [years]**, mean (SD)		32 (13)	35 (14)	0.202
	missing (n)	-	89	
**Time to first assessment (days)**, mean (SD)		9 (15)	10 (13)	0.165
	missing (n)	-	89	
**Time to first follow-up (days)**, mean (SD)		209 (55)	210 (55)	0.642
	missing (n)		176	
**Sex (female)**, n (%)		122 (20.8)	19 (9.0)	0.250
	missing (n)	-	89	
**VAC (yes)**, n (%)		87 (14.8)	20 (9.5)	0.735
	missing (n)	-	89	
**DAP (yes)**, n (%)		202 (34.4)	52 (24.8)	0.092

S3B		Sygen (n=797)		
		**benchmark** (n=587)	**Excluded** (n=210)	**p-value**
	missing (n)	-	89	
**Neurological level of injury**, n (%)				
	cervical	448 (76.3)	103 (49.0)	0.045
	thoracic	139 (23.7)	18 (8.6)	
	missing	-	89	
**Injury severity (AIS grade)**, n (%)				
	AIS A	377 (64.2)	67 (32.0)	0.302
	AIS B	64 (10.9)	16 (7.6)	
	AIS C	118 (20.1)	32 (15.2)	
	AIS D	28 (4.8)	6 (2.9)	
	missing	-	89	

American Spinal Injury Association Impairment Scale (AIS): AIS-A no sensory or motor function is preserved in the sacral segments S4-5. AIS-B sensory but no motor function is preserved below the neurological level and includes the sacral segments S4-5 (LT or PP at S4-5 or DAP), and no motor function is preserved more than three levels below the motor level on either side of the body. AIS-C motor function is preserved at the most caudal sacral segments for voluntary anal contraction OR the patient meets the criteria for sensory incomplete status, and has some sparing of motor function more than three levels below the ipsilateral motor level on either side of the body. Less than half of key muscle functions below the single NLI have a muscle grade ≥ 3. AIS-D motor incomplete status as defined above, with at least half (half or more) of key muscle functions below the single NLI having a muscle grade ≥ 3. AIS-E if sensation and motor function as tested with the ISNCSCI are graded as normal in all segments, and the patient had prior deficits, then the AIS grade is E. Someone without an initial SCI does not receive an AIS grade. VAC: voluntary anal contraction. DAP: deep anal pressure.

2.2.2. Imputation

Age was missing for 35 patients in the EMSCI cohort, for whom sufficient information was otherwise available to be included in the analysis, resulting in 127 instances with imputed age values (see **Supplementary Table S4**). These missing values were replaced with the mean age (46, see **Table 1**). **Supplementary Tables S2** and **S3** show the number of instances for which VAC and DAP were imputed within each AIS grade. In Sygen, 0 patients were missing age, 3 patients were missing VAC (all with AIS C and imputed with the mode of the AIS grade: 'Yes'), and 8 patients were missing DAP (all with AIS C and imputed 'Yes').

Supplementary Table S4: Number of instances in EMSCI with missing age.

**Initial exam stage**	**Final exam stage**	**Number of patients with missing age**
Very acute	Acute III	14
Very acute	Chronic	11
Acute I	Acute III	29
Acute I	Chronic	22
Acute II	Acute III	29
Acute II	Chronic	22

Supplementary Table S5: Number of instances in EMSCI within each AIS grade with imputed VAC. Imputation was performed using the mode for each AIS grade (A, B, C - No, D - Yes).

Initial exam stage	Final exam stage	AIS A	AIS B	AIS C	AIS D
Very acute	Acute III	0	0	12	24
Very acute	Chronic	0	0	16	29
Acute I	Acute III	0	0	19	37
Acute I	Chronic	0	0	24	48
Acute II	Acute III	0	0	15	46
Acute II	Chronic	0	0	16	47

Supplementary Table S6: Number of instances in EMSCI within each AIS grade with imputed DAP. Imputation was performed using the mode for each AIS grade (A - No, B, C, D - Yes).

Initial exam stage	Final exam stage	AIS A	AIS B	AIS C	AIS D
Very acute	Acute III	0	2	12	24
Very acute	Chronic	0	3	16	29
Acute I	Acute III	0	4	16	36
Acute I	Chronic	0	4	21	45
Acute II	Acute III	0	3	12	39
Acute II	Chronic	0	2	13	45

2.2. Model benchmark

We compare SCI recovery prediction performance using $RMSE_{bl.NLI}$ and mean residuals below the NLI in **Tables S7-12**. There is a small performance deterioration across all models in Sygen compared to EMSCI, leading to a median $RMSE_{bl.NLI}$ of 0.57 for the best-performing model (CNN), and a full range of median $RMSE_{bl.NLI}$ of 0.57-0.71 for Sygen compared to 0.55-0.64 for EMSCI. Although we ensured a comparable window of input data acquisitions (4±1 weeks), differences in cohort characteristics, such as patient age and injury severity distributions, remained.

Supplementary Table S7: Results of the model benchmark. Values shown are median $RMSE_{bl.NLI}$ (lower values are better) and (2.5%-ile, 97.5%-ile) on test sets for EMSCI and Sygen cohorts (external validation). The best-performing approach (lowest $RMSE_{bl.NLI}$, if tied on median lower value for 97.5%-ile better) is highlighted in bold. Columns labeled *all times* include all instances, while columns labeled *4±1 weeks* include only instances for which the initial assessment was between 21 and 35 days after injury.

	**EMSCI**		**Sygen**	
	All times	4±1 weeks	All times	4±1 weeks
**XGBoost**	0.61 (0.01, 2.12)	0.61 (0.01, 2.08)	0.70 (0.01, 2.33)	0.68 (0.02, 2.00)
**Random Forest**	0.61 (0.01, 2.17)	0.60 (0.01, 2.08)	0.64 (0.03, 2.37)	0.61 (0.03, 1.89)
**Random Forest (MS same)**	0.64 (0.00, 2.21)	0.62 (0.02, 2.13)	0.69 (0.00, 2.24)	0.67 (0.09, 2.01)
**Ridge**	0.64 (0.00, 2.27)	0.65 (0.00, 2.19)	0.71 (0.00, 2.42)	0.72 (0.07, 2.26)
**ElasticNet**	0.63 (0.00, 2.22)	0.64 (0.02, 2.15)	0.70 (0.00, 2.32)	0.68 (0.08, 2.15)
**CNN (multi-modal)**	**0.55** (0.00, 2.50)	**0.55** (0.00, 2.57)	0.57 (0.00, 2.85)	**0.60** (0.00, 2.90)
**CNN (ensemble)**	0.55 (0.00, 2.51)	0.56 (0.00, 2.53)	**0.57** (0.00, 2.77)	0.63 (0.00, 2.85)
**CNN (block)**	0.57 (0.00, 2.65)	0.59 (0.00, 2.52)	0.63 (0.00, 2.82)	0.69 (0.00, 2.86)
**Transformer (multi-modal)**	0.63 (0.00, 2.58)	0.63 (0.00, 2.57)	0.65 (0.00, 3.00)	0.71 (0.00, 3.27)
**Transformer (block)**	0.63 (0.00, 2.86)	0.63 (0.00, 2.83)	0.67 (0.00, 2.72)	0.73 (0.00, 2.66)
**GNN**	0.58 (0.00, 2.60)	0.60 (0.00, 2.47)	0.68 (0.00, 2.61)	0.63 (0.00, 2.57)

Abbreviations: $RMSE_{bl.NLI}$: Root mean squared error (RMSE) below neurological level of injury (blNLI); CNN: convolutional neural network; GNN: graph neural network.

Supplementary Table S8: Performance (median $RMSE_{bl.NLI}$) on the EMSCI dataset stratified by AIS grade. Percentiles 2.5 and 97.5 are shown in parentheses.

	**AIS A**	**AIS B**	**AIS C**	**AIS D**
**XGBoost**	0.31 (0.00, 1.89)	0.97 (0.20, 2.76)	1.17 (0.51, 2.35)	0.57 (0.14, 1.43)
**Random Forest**	0.28 (0.00, 1.92)	0.98 (0.18, 2.87)	1.21 (0.53, 2.39)	0.58 (0.19, 1.52)
**Random Forest (MS same)**	0.30 (0.00, 1.86)	0.99 (0.18, 2.82)	1.24 (0.54, 2.32)	0.58 (0.15, 1.58)
**Ridge**	0.36 (0.00, 2.03)	0.95 (0.11, 3.18)	1.24 (0.53, 2.48)	0.62 (0.00, 1.56)
**ElasticNet**	0.36 (0.00, 1.97)	0.92 (0.10, 3.09)	1.22 (0.56, 2.43)	0.62 (0.00, 1.52)
**CNN (multimodal)**	0.04 (0.00, 2.23)	0.78 (0.00, 3.31)	**1.16** (0.27, 2.80)	0.60 (0.00, 1.65)
**CNN (ensemble)**	0.03 (0.00, 2.27)	**0.76** (0.00, 3.38)	1.17 (0.27, 2.78)	0.60 (0.00, 1.60)
**CNN (block)**	**0.00** (0.00, 2.24)	0.88 (0.00, 3.74)	1.24 (0.31, 3.10)	0.60 (0.00, 1.61)
**Transformer (multi-modal)**	0.02 (0.00, 2.21)	0.94 (0.00, 3.52)	1.25 (0.41, 2.89)	0.66 (0.00, 1.70)
**Transformer (block)**	0.00 (0.00, 2.34)	0.99 (0.00, 3.72)	1.38 (0.40, 3.24)	0.66 (0.00, 1.79)
**GNN**	0.06 (0.00, 2.11)	0.90 (0.01, 3.54)	1.27 (0.39, 2.89)	**0.58** (0.01, 1.57)

Abbreviations: $RMSE_{bl.NLI}$: Root mean squared error (RMSE) below neurological level of injury (blNLI); AIS: American Spinal Injury Association (ASIA) Injury Severity; CNN: convolutional neural network; GNN: graph neural network.

Supplementary Table S9: Performance (median $RMSE_{bl.NLI}$) on the Sygen dataset stratified by AIS grade. Percentiles 2.5 and 97.5 are shown in parentheses.

	**AIS A**	**AIS B**	**AIS C**	**AIS D**
**XGBoost**	0.51 (0.00, 1.91)	1.39 (0.50, 3.16)	1.08 (0.47, 2.42)	0.53 (0.13, 1.32)
**Random Forest**	0.44 (0.00, 1.85)	**1.25** (0.50, 3.33)	1.14 (0.49, 2.49)	0.61 (0.16, 1.41)
**Random Forest (MS same)**	0.47 (0.00, 1.86)	1.29 (0.51, 3.22)	1.17 (0.56, 2.40)	0.57 (0.12, 1.39)
**Ridge**	0.54 (0.00, 1.98)	1.34 (0.51, 3.51)	1.20 (0.23, 2.52)	0.66 (0.00, 1.75)
**ElasticNet**	0.53 (0.00, 1.89)	1.29 (0.51, 3.52)	1.18 (0.23, 2.38)	0.65 (0.00, 1.52)
**CNN (multi-modal)**	**0.38** (0.00, 2.61)	1.51 (0.25, 3.85)	**1.05** (0.00, 3.14)	**0.45** (0.00, 1.76)
**CNN (ensemble)**	0.38 (0.00, 2.73)	1.47 (0.31, 3.85)	1.07 (0.00, 2.90)	0.47 (0.00, 1.91)
**CNN (block)**	0.39 (0.00, 2.65)	1.39 (0.33, 4.08)	1.12 (0.00, 3.39)	0.52 (0.00, 2.58)
**Transformer (multi-modal)**	0.41 (0.00, 3.03)	1.43 (0.34, 4.08)	1.17 (0.15, 3.13)	0.52 (0.00, 1.40)
**Transformer (block)**	0.42 (0.00, 2.81)	1.56 (0.34, 4.10)	1.18 (0.08, 3.30)	0.55 (0.00, 1.73)
**GNN**	0.45 (0.00, 2.10)	1.66 (0.42, 3.61)	1.18 (0.32, 2.63)	0.56 (0.08, 1.40)

Abbreviations: $RMSE_{bl.NLI}$: Root mean squared error (RMSE) below neurological level of injury (blNLI); AIS: American Spinal Injury Association (ASIA) Injury Severity; CNN: convolutional neural network; GNN: graph neural network.

Supplementary Table S10: Median of mean residuals below the NLI (predictions subtracted from ground truth, i.e. values smaller than zero indicate observed recovery worse than predicted). Numbers are written as median (2.5%, 97.5%). Values closer to 0.00 are better.

	**EMSCI**		**Sygen**	
	all times	4±1 weeks	all times	4±1 weeks
**XGBoost**	-0.02 (-1.19, 1.55)	-0.02 (-1.21, 1.51)	-0.04 (-1.47, 1.62)	-0.04 (-1.40, 1.31)
**Random Forest**	-0.04 (-1.20, 1.71)	-0.02 (-1.21, 1.58)	-0.04 (-1.11, 1.96)	-0.04 (-1.20, 1.37)
**Random Forest (MS same)**	-0.04 (-1.28, 1.59)	-0.12 (-1.36, 1.47)	-0.15 (-1.22, 1.72)	-0.18 (-1.28, 1.19)
**Ridge**	-0.02 (-1.15, 1.80)	-0.10 (-1.18, 1.70)	-0.15 (-1.17, 1.92)	-0.18 (-1.20, 1.47)
**ElasticNet**	-0.03 (-1.12, 1.77)	-0.12 (-1.15, 1.71)	-0.14 (-1.14, 1.88)	-0.19 (-1.19, 1.48)
**CNN multi-modal**	0.00 (-1.41, 1.80)	0.00 (-1.37, 1.94)	0.00 (-1.66, 1.88)	0.00 (-1.56, 1.77)
**CNN (ensemble)**	0.00 (-1.26, 1.91)	0.00 (-1.23, 2.00)	0.00 (-1.57, 1.89)	0.00 (-1.64, 1.75)
**CNN (block)**	0.00 (-1.44, 1.87)	0.00 (-1.48, 1.72)	0.00 (-1.27, 2.00)	0.00 (-1.48, 1.63)
**Transformer**	0.00 (-1.24, 1.90)	0.00 (-1.31, 1.87)	0.00 (-1.92, 1.92)	0.00 (-2.54, 1.57)
**Transformer (block)**	0.00 (-1.55, 2.03)	0.00 (-1.51, 2.05)	0.00 (-1.64, 2.01)	0.00 (-1.85, 1.57)
**GNN**	**0.00 (-1.30, 1.74)**	**0.00 (-1.32, 1.72)**	**0.00 (-1.30, 1.68)**	**0.00 (-1.21, 1.63)**

Abbreviations: NLI: neurological level of injury; CNN: convolutional neural network; GNN: graph neural network.

Supplementary Table S11: Median of mean residual below NLI on the EMSCI dataset stratified by AIS grade. Percentiles 2.5 and 97.5 are shown in parentheses.

	AIS A	AIS B	AIS C	AIS D
**XGBoost**	-0.03 (-0.77, 1.19)	-0.24 (-1.70, 2.48)	-0.03 (-1.58, 1.92)	0.09 (-0.82, 0.75)
**Random Forest**	-0.06 (-0.65, 1.31)	-0.32 (-1.61, 2.63)	-0.07 (-1.58, 2.12)	0.11 (-0.97, 0.85)
**Random Forest (MS same)**	-0.08 (-0.72, 1.18)	-0.30 (-1.64, 2.78)	-0.20 (-1.82, 1.95)	0.07 (-0.90, 0.83)
**Ridge**	-0.06 (-0.69, 1.47)	-0.21 (-1.33, 2.87)	-0.09 (-1.60, 2.15)	0.05 (-0.76, 0.95)
**ElasticNet**	-0.07 (-0.67, 1.42)	-0.21 (-1.31, 2.85)	-0.10 (-1.58, 2.10)	0.05 (-0.74, 0.96)
**CNN multi-modal**	0.00 (-0.51, 1.66)	**0.00 (-1.71, 2.81)**	-0.13 (-2.02, 2.06)	-0.17 (-1.25, 0.55)
**CNN (ensemble)**	0.00 (-0.46, 1.66)	0.00 (-1.69, 3.10)	**0.00 (-1.94, 2.18)**	-0.12 (-1.15, 0.74)
**CNN (block)**	0.00 (-0.52, 1.52)	0.00 (-2.13, 2.99)	0.00 (-2.16, 2.34)	-0.08 (-1.10, 0.76)
**Transformer**	0.00 (-0.53, 1.54)	0.01 (-2.23, 3.15)	0.10 (-1.86, 2.25)	0.01 (-0.96, 1.10)
**Transformer (block)**	0.00 (-0.76, 1.57)	0.00 (-2.49, 3.13)	0.00 (-2.47, 2.50)	-0.04 (-1.14, 1.00)
**GNN**	**0.00 (-0.52, 1.41)**	-0.02 (-2.18, 2.93)	-0.01 (-1.95, 2.08)	**-0.01 (-0.99, 0.77)**

Abbreviations: NLI: neurological level of injury; AIS: American Spinal Injury Association (ASIA) Injury Severity; CNN: convolutional neural network; GNN: graph neural network.

Supplementary Table S12: Median of mean residual below NLI on the Sygen dataset stratified by AIS grade. Percentiles 2.5 and 97.5 are shown in parentheses.

	AIS A	AIS B	AIS C	AIS D
XGBoost	-0.08 (-1.00, 0.92)	-0.20 (-2.14, 2.70)	0.39 (-1.92, 1.73)	0.15 (-0.85, 0.66)
Random Forest	-0.06 (-0.79, 1.05)	-0.08 (-1.61, 3.19)	0.51 (-1.39, 2.21)	0.21 (-0.75, 1.03)
Random Forest (MS same)	-0.21 (-0.93, 0.88)	-0.27 (-1.71, 3.21)	0.27 (-1.54, 1.94)	0.07 (-0.73, 1.03)
Ridge	-0.21 (-0.93, 1.09)	-0.07 (-1.59, 3.27)	0.23 (-1.48, 2.13)	0.06 (-0.70, 1.29)
ElasticNet	-0.22 (-0.91, 1.04)	-0.07 (-1.50, 3.32)	0.24 (-1.45, 2.12)	0.01 (-0.68, 1.27)
CNN (multi-modal)	0.00 (-0.70, 1.32)	0.12 (-2.78, 2.80)	-0.02 (-2.42, 1.60)	-0.09 (-1.04, 0.66)
CNN (ensemble)	0.00 (-0.91, 1.42)	0.16 (-2.39, 2.99)	**0.00 (-2.28, 1.68)**	-0.06 (-0.95, 0.91)
CNN (block)	0.00 (-0.88, 1.36)	0.06 (-1.79, 3.59)	0.15 (-2.02, 2.15)	-0.03 (-0.91, 1.24)
Transformer (multi-modal)	0.00 (-1.84, 1.25)	0.13 (-2.21, 3.71)	0.32 (-1.95, 2.05)	**0.00 (-0.55, 0.42)**
Transformer (block)	0.00 (-1.35, 1.23)	0.07 (-2.18, 3.35)	0.13 (-1.84, 2.09)	-0.01 (-0.93, 0.61)
GNN	**0.00 (-0.66, 1.19)**	**0.02 (-3.00, 2.55)**	0.21 (-1.94, 1.61)	0.05 (-0.65, 0.78)

Abbreviations: NLI: neurological level of injury; AIS: American Spinal Injury Association (ASIA) Injury Severity; CNN: convolutional neural network; GNN: graph neural network.

Supplementary Table S13: Benchmark on EMSCI cohort with NISCI inclusion criteria (UEMS at baseline>29, age 18-70, baseline ISNCSCI at 4-28 days, follow-up at 150-210 days, and cervical injury). Median difference in UEMS at follow-up and prediction, median $RMSE_{bl.NLI.}$, and median of mean residual below NLI. Percentiles 2.5 and 97.5 are shown in parentheses.

	UEMS	RMSEbl.NLI.	Residual below NLI
XGBoost	7.58 (2.39, 19.30)	0.93 (0.26, 2.48)	-0.05 (-1.45, 2.04)
Random Forest	7.66 (2.69, 19.57)	0.93 (0.29, 2.60)	-0.03 (-1.38, 2.12)
Random Forest (MS same)	7.81 (3.38, 18.87)	0.94 (0.36, 2.49)	-0.12 (-1.37, 2.18)
Ridge	8.11 (2.89, 20.16)	0.98 (0.35, 2.69)	-0.08 (-1.23, 2.30)
ElasticNet	7.95 (3.29, 19.13)	0.94 (0.37, 2.63)	-0.09 (-1.21, 2.24)
CNN multi-modal	6.98 (0.93, 20.83)	0.93 (0.14, 2.93)	-0.06 (-1.75, 2.35)
CNN (ensemble)	**6.88 (0.86, 21.09)**	**0.90 (0.15, 2.95)**	-0.03 (-1.56, 2.41)
CNN (block)	7.10 (0.54, 23.01)	0.96 (0.11, 3.10)	**0.02 (-1.61, 2.44)**
Transformer	7.42 (0.55, 23.19)	0.96 (0.11, 3.14)	0.07 (-1.36, 2.64)
Transformer (block)	7.86 (0.45, 23.42)	1.04 (0.08, 3.15)	0.06 (-1.87, 2.37)

Supplementary Table S14: Benchmark of CNN multi-modal trained on the same data as before and all combinations of timepoints before, including the prediction time being the same as the input time. The evaluation times in the first row are the same as in e.g. Table S7. Note that this model slightly outperformed the same model trained on the standard data times.

	**EMSCI**		**Sygen**	
	All times	4±1 weeks	All times	4±1 weeks
CNN multi-modal	0.54 (0.00, 2.47)	0.55 (0.00, 2.58)	0.57 (0.00, 2.72)	0.59 (0.00, 2.66)
**Evaluated also on early time points**				
CNN multi-modal	0.24 (0.00, 1.88)	0.28 (0.00, 1.95)		

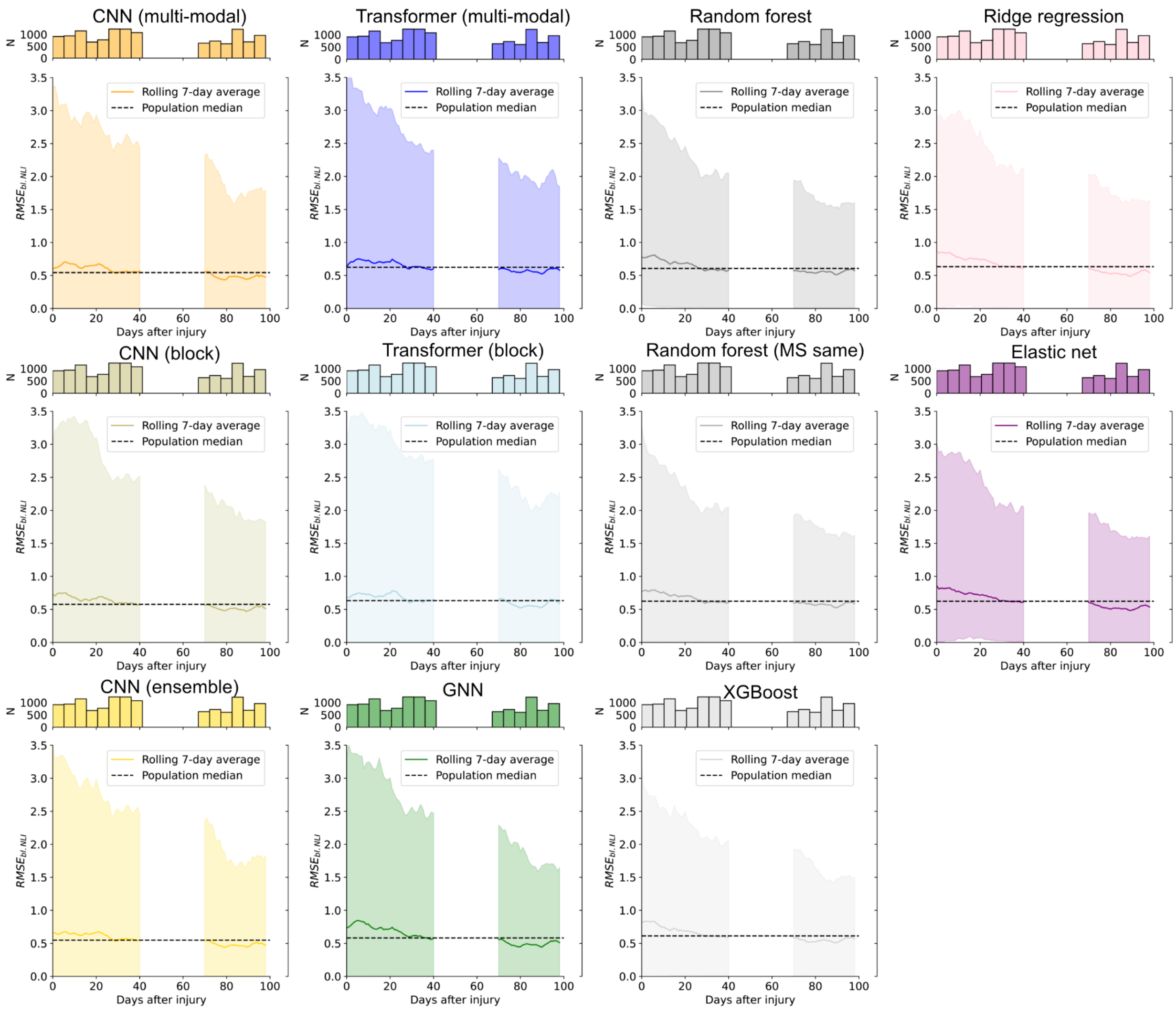

Supplementary Figure S4: **$RMSE_{bl.NLI}$ as function of time of initial assessment** in days after injury for all eleven tested models. The dashed line shows the 7-day rolling mean of the medians for each day and the shade is the 2.5th-97.5th percentile. Histograms indicate the number N of patient data points supporting the rolling mean.

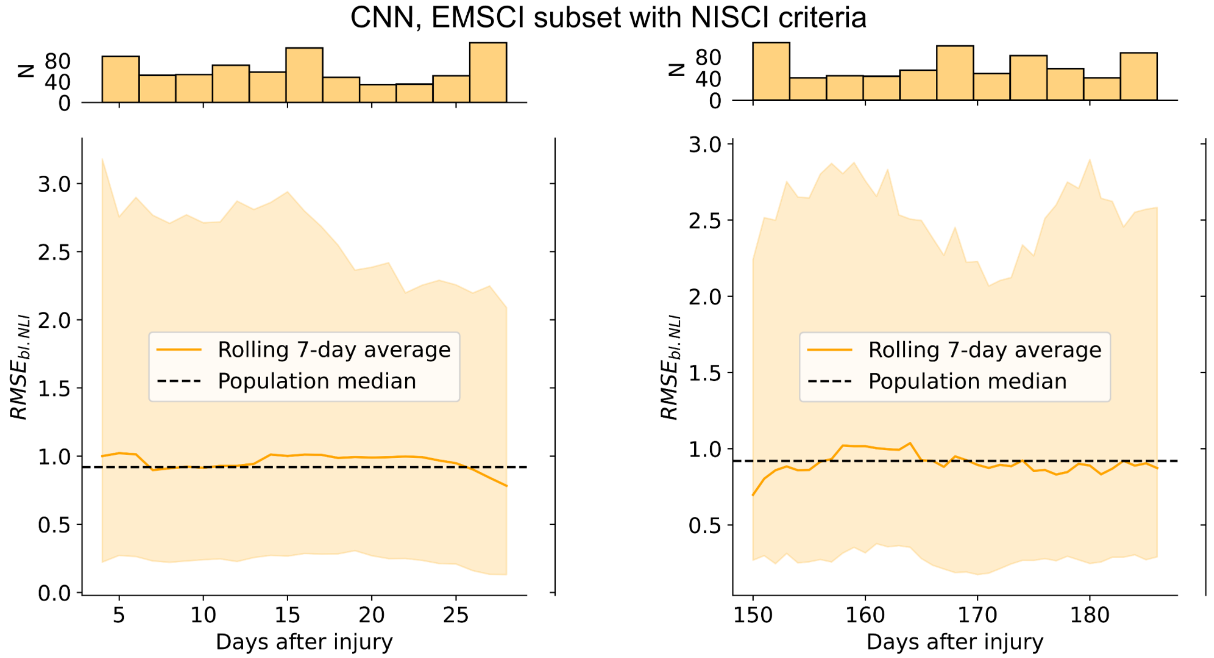

Supplementary Figure S5: **RMSE$_{bl.NLI}$ over time of initial assessment** in days after injury for the CNN, evaluated on the EMSCI patients that follow the NISCI inclusion criteria (UEMS at baseline>29, age 18-70, baseline ISNCSCI at 4-28 days, follow-up at 150-210 days, and cervical injury). The dashed line shows the 7-day rolling mean of the medians for each day and the shade is the 2.5th-97.5th percentile. Histograms indicate the number N of patient data points supporting the rolling mean.

2.3 Interpretability analysis

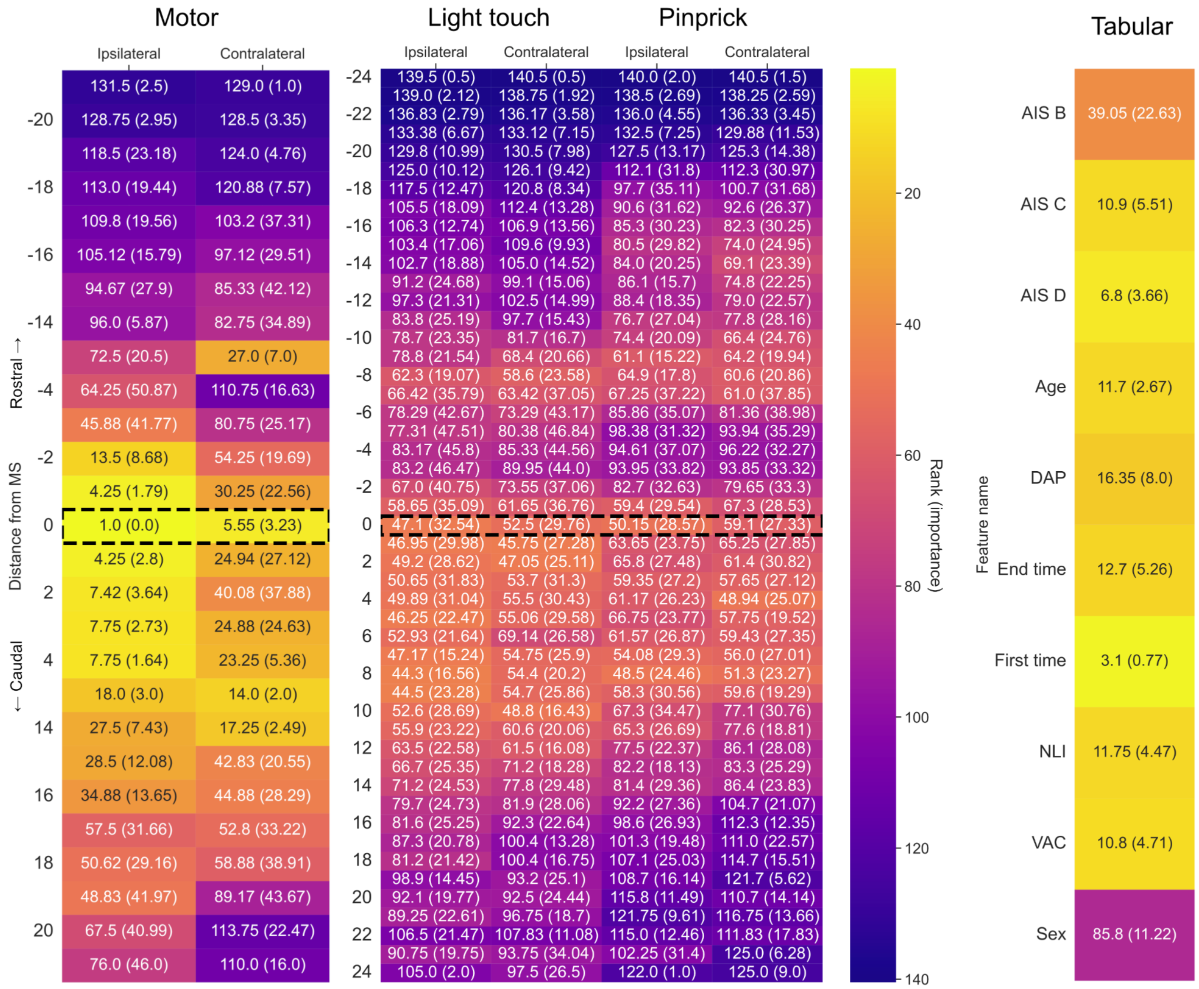

Supplementary Figure S6: Importance ranking of interpretability SHAP scores in relation to the endpoint MS, aggregated over all MS. The annotated values are mean rank values (standard deviation) using the CNN model. The colors are by rank (same as Figure 2H, and not by mean rank value as the annotated value. Note that AIS A is not present as its own feature category in one-hot encoding.

2.4 Trial simulations

Supplementary Table S15: Distribution of group-level differences in mean $LEMS_{impr.}$ ($\Delta LEMS_{impr.}$) for randomized and synthetic controls originating from different models also shown in Figure 3C. CNN: convolutional neural network; GNN: graph neural network.

**control**	**median**	**25%-ile**	**75%-ile**
randomized	0.035	-0.975	0.878
synthetic (linear)	0.928	0.301	1.529
synthetic (XGBoost)	0.514	0.007	1.058
synthetic (random forest)	1.537	0.934	2.095
synthetic (CNN)	0.106	-0.577	0.658
synthetic (transformer)	1.042	0.326	1.671
synthetic (GNN)	0.734	0.149	1.389

2.5 Case study

The outcome ($UEMS_{impr.}$) observed in the placebo group of the NISCI trial equals 12.67 [1.00, 26.85] (mean [2.5%-ile, 97.5%-ile]) while equivalent values for the outcome estimated from the synthetic controls equals 14.05 [1.93, 24.12] for the placebo group and 13.42 [0.13, 26.26] for the treatment group. Differences are not statistically significant (placebo observed vs. placebo synthetic controls: $p=0.176$; placebo observed vs. treatment synthetic controls: $p=0.582$), suggesting that the synthetic controls provide a reasonable counterfactual in the context of the NISCI trial. The relevant values of the outcome measure of interest ($UEMS_{impr.}$) used for the power calculation are $\mu=\mu_O=12.792$, $\mu_R=12.309$, $\sigma=9.813$, and $u=8.373$.
